# Graphene-based FETs for advanced biocatalytic profiling: investigating heme peroxidase activity with machine learning insights

**DOI:** 10.1007/s00604-025-06955-y

**Published:** 2025-03-03

**Authors:** Samaneh Mirsian, Wolfgang Hilber, Ehsan Khodadadian, Maryam Parvizi, Amirreza Khodadadian, Seyyed Mehdi Khoshfetrat, Clemens Heitzinger, Bernhard Jakoby

**Affiliations:** 1 Institute of Microelectronics and Microsensors, Johannes Kepler University, Linz, Austria; 2 Research Unit Machine Learning, Institute of Information Systems Engineering, Department of Informatics, TU Wien, Vienna, Austria; 3 School of Mathematics, University of Birmingham, Birmingham, UK; 4 School of Computer Science and Mathematics, Keele University, Newcastle-under-Lyme, UK; 5 Department of Chemistry, Faculty of Basic Sciences, Ayatollah Boroujerdi University, Boroujerd, Iran

**Keywords:** Biocatalytic, Enzyme, Graphene field-effect transistors, Heme peroxidases, Deep neural networks

## Abstract

**Abstract:**

Graphene-based field-effect transistors (GFETs) are rapidly gaining recognition as powerful tools for biochemical analysis due to their exceptional sensitivity and specificity. In this study, we utilize a GFET system to explore the peroxidase-based biocatalytic behavior of horseradish peroxidase (HRP) and the heme molecule, the latter serving as the core component responsible for HRP’s enzymatic activity. Our primary objective is to evaluate the effectiveness of GFETs in analyzing the peroxidase activity of these compounds. We highlight the superior sensitivity of graphene-based FETs in detecting subtle variations in enzyme activity, which is critical for accurate biochemical analysis. Using the transconductance measurement system of GFETs, we investigate the mechanisms of enzymatic reactions, focusing on suicide inactivation in HRP and heme bleaching under two distinct scenarios. In the first scenario, we investigate the inactivation of HRP in the presence of hydrogen peroxide and ascorbic acid as cosubstrate. In the second scenario, we explore the bleaching of the heme molecule under conditions of hydrogen peroxide exposure, without the addition of any cosubstrate. Our findings demonstrate that this advanced technique enables precise monitoring and comprehensive analysis of these enzymatic processes. Additionally, we employed a machine learning algorithm based on a multilayer perceptron deep learning architecture to detect the enzyme parameters under various chemical and environmental conditions. Integrating machine learning and probabilistic methods significantly enhances the accuracy of enzyme behavior predictions.

**Graphical abstract:**

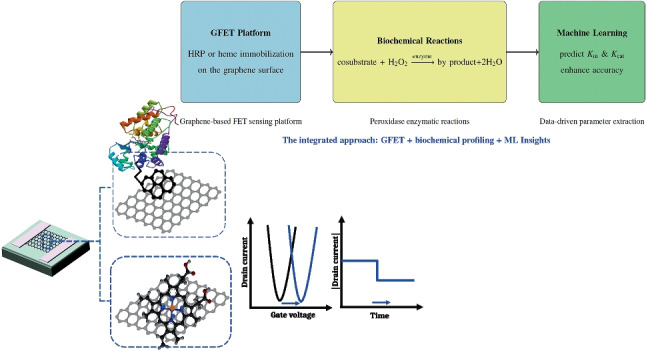

**Supplementary Information:**

The online version contains supplementary material available at 10.1007/s00604-025-06955-y.

## Introduction

Organisms continuously adapt to genetic and environmental changes, requiring a deep understanding of biochemical and biophysical networks in metabolism. Enzymes, which drive most of these metabolic processes, must be studied in detail to understand how environmental perturbations affect complex metabolic reaction networks. Kinetic models have been vital for over 50 years in exploring enzyme mechanisms and metabolic networks, impacting fields like synthetic biology and biotechnology [1]. Apart from this, enzymes, including hydrolases and redox enzymes, are crucial in industrial processes. Heme-iron oxidases, such as peroxidases, are used in applications ranging from enzymatic scavenging to biofuel production [2]. However, peroxidases often suffer from suicide inactivation by hydrogen peroxide (H$_2$O$_2$), which can lead to enzyme deactivation through biliverdin formation or heme destruction [3, 4]. High H$_2$O$_2$ concentrations complicate enzyme activity assessments, and a variety of techniques and different strategies have been applied to study this phenomenon.

Morales-Urrea et al. used UV–Vis spectroscopy to study HRP inactivation by H$_2$O$_2$, revealing enzyme deactivation through heme degradation. They identified three states of HRP: resting (E$_1$), low H$_2$O$_2$ (E$_2$), and high H$_2$O$_2$ (E$_3$), with E$_3$ decaying to E$_0$ and forming a new inactive species with an absorption band at 670 nm. External substrates prevent E$_3$ formation by accelerating H$_2$O$_2$ consumption [2]. Khosraneh et al. studied microperoxidase-11 (MP-11) reacting with H$_2$O$_2$ to oxidize guaiacol using UV–Vis spectroscopy, confirming first-order kinetics with respect to guaiacol. Their results suggest MP-11 inactivation by suicide-peroxide is similar to HRP, with inactivation occurring even at low H$_2$O$_2$ (0.4 mM) [5]. Savéant et al. explored HRP’s mechanisms involving H$_2$O$_2$ and an electron donor, finding a bell-shaped calibration curve using the cyclic voltammetry method. This was attributed to the formation of inactive oxyperoxidase, and they demonstrated that at low H$_2$O$_2$ concentrations, the response is diffusion-controlled and proportional to H$_2$O$_2$ levels [6].

To address enzyme structural changes, molecular biologists employ techniques to enhance stability, such as protecting oxidation-sensitive residues or optimizing free radical pathways. These advances improve enzyme longevity and utility across various applications [4]. However, challenges like instability and high costs have driven interest in synthetic enzyme mimics, such as nanozymes, which offer precise control over enzyme-driven reactions. These materials provide tailored solutions for catalytic applications in biotechnology, medicine, and environmental preservation [7–10].

Single-atom nanozymes have the potential to revolutionize artificial enzymes by precisely manipulating their active site properties, offering a promising alternative to natural enzymes. These nanozymes bridge heterogeneous, homogeneous, and enzymatic catalysis, advancing catalytic technology [11]. Notably, the heme molecule is often emulated in their design, with enzymatic activity typically assessed by comparing their interaction with H$_2$O$_2$. However, the specific mechanism of suicide inactivation related to the heme molecule remains unclear.

FETs as electronic devices offer exceptional real-time responsiveness and sensitivity for studying enzymatic reactions. GFETs excel in this domain due to their remarkable sensitivity and ability to monitor enzyme activity in real time. GFETs enable label-free detection by directly observing changes in electrical properties, providing quantitative insights into reaction kinetics and mechanisms. Their adaptability allows for selective interactions with specific enzymes or reaction products, and when integrated with microfluidic devices, they offer precise control over reaction conditions. GFETs are particularly valuable for studying redox-active enzymes like HRP and heme, optimizing substrate concentrations, minimizing enzyme inactivation, and providing critical insights into structural changes during enzymatic reactions.

In recent years, a variety of GFETs have been developed for enzymatic biosensing, demonstrating their potential for highly sensitive detection of small molecules. Wang et al. introduced a photoelectrochemical solution-gated graphene FET (PEC-SGGT) that integrates enzyme cascade reactions for organophosphorus detection, achieving a thousandfold current gain and a low detection limit of $0.05\,\textrm{pM}$ [12]. Fenoy et al. developed a method for immobilizing acetylcholinesterase on graphene FETs, enhancing pH sensitivity and achieving a detection range for acetylcholine from $5\,\mathrm {\mu M}$ to $1000\,\mathrm {\mu M}$ [13]. Piccini et al. demonstrated the construction of urea-sensing biosensors using reduced graphene oxide FETs, with a low detection limit of $1\,\mathrm {\mu M}$ and the ability to quantify Cu$^{2+}$ through urease inhibition [14]. You et al. created a silk fibroin-encapsulated graphene FET biosensor for glucose detection, demonstrating a linear detection range from $0.1\,\textrm{mM}$ to $10\,\textrm{mM}$ [15]. Kwak et al. designed a flexible glucose sensor using CVD-grown graphene FETs, capable of real-time monitoring with a detection range of $3.3\,\textrm{mM}$-$10.9\,\textrm{mM}$ [16]. Furthermore, Wei et al. developed a heat-denatured casein-modified graphene FET biosensor for the ultrasensitive detection of $\beta$-galactosidase, achieving an attomole sensitivity and a detection range of $1\,\mathrm {fg/mL}$-$100\,\mathrm {ng/mL}$ [17]. These studies underscore the versatility and sensitivity of GFETs for enzymatic biosensing applications. However, to the best of our knowledge, none of these investigations have utilized GFET systems for a detailed exploration of enzymatic reaction mechanisms.

Enzyme kinetic parameters (Michaelis-Menten (MM) parameters), are fundamental for describing how enzymes interact with substrates and catalyze reactions. They quantitatively define substrate affinity and the maximum catalytic rate, enabling precise modeling of enzymatic behavior under various conditions. Accurately determining MM parameters experimentally presents significant challenges. These include the complexity of biological systems with potential for enzyme impurities and the presence of inhibitors, stringent experimental requirements for maintaining constant conditions and accurate measurements, and inherent limitations of the MM model itself, such as the steady-state assumption and its applicability to single-substrate reactions [18]. Furthermore, environmental factors such as pH and temperature can significantly influence enzyme activity, further complicating the process [19].

**Fig. 1:**
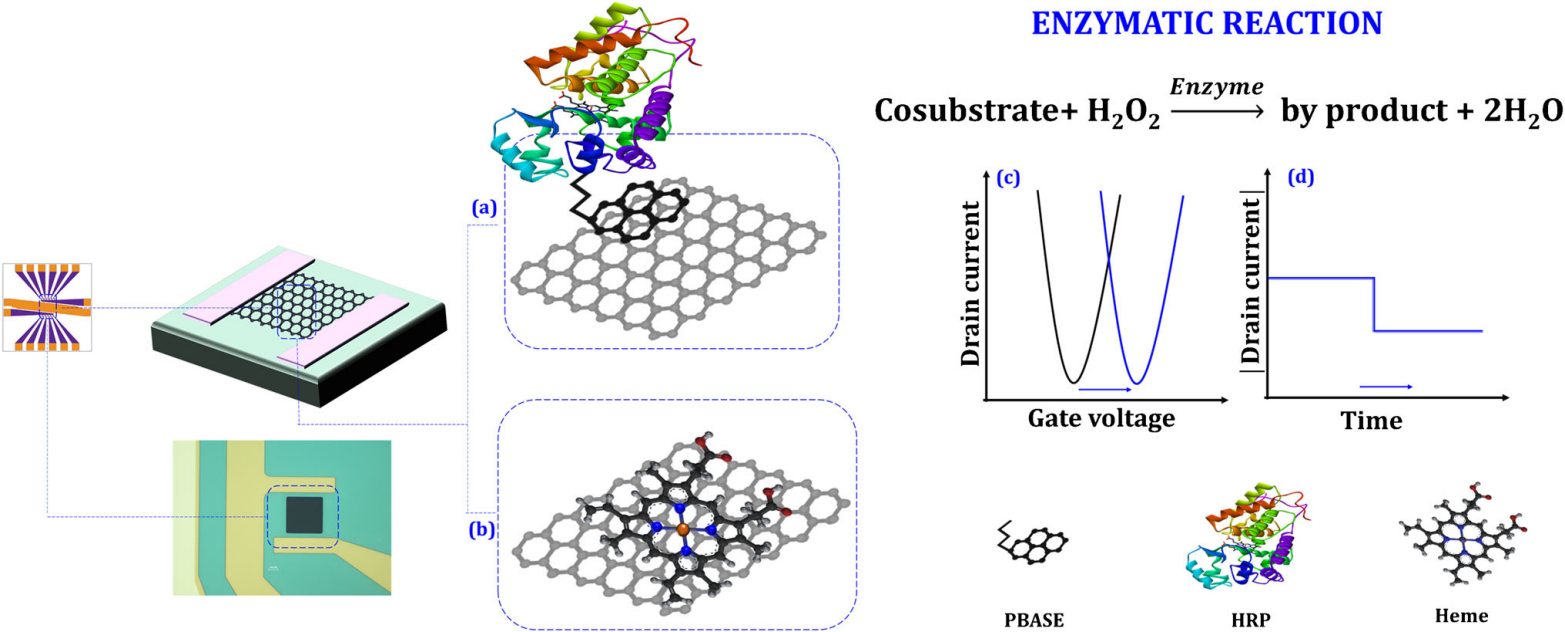
A graphical summary of the experimental procedures employed in this study includes the following steps: **a** HRP immobilization on graphene: HRP is immobilized on the graphene surface using PBASE as a cross-linker; **b** heme immobilization on graphene. Enzymatic activity is assessed through two methods: **c** transconductance measurement: the $I_{\textrm{ds}}$ current is recorded while varying $V_{\textrm{g}}$ at a fixed $V_{\textrm{ds}}$; and **d** constant voltage mode: MM parameters are determined by measuring $I_{\textrm{ds}}$ at constant $V_{\textrm{ds}}$ and $V_{\textrm{g}}$

Bayesian inversion and machine learning offer significant advantages over traditional experimental methods for estimating MM parameters. Bayesian inversion [20, 21] employs a probabilistic framework to integrate prior knowledge and experimental data, enabling robust parameter estimation while effectively managing uncertainties and model discrepancies [20, 22, 23]. For example, Choi et al. [24] demonstrated its efficacy in identifying MM parameters ($K_\textrm{M}$, $k_{\text {cat}}$) using ODE-based models and MCMC methods. Machine learning further enhances parameter prediction through algorithms like regression and neural networks, offering improved accuracy and efficiency for different environmental and chemical conditions. Maeda et al. [25] successfully applied global optimization-based machine learning to estimate the MM parameters. Unlike labor-intensive experimental techniques, these computational methods reduce reliance on extensive testing, handle complex biochemical scenarios, and provide scalable and reliable parameter estimation. By combining Bayesian rigor with machine learning’s predictive capabilities, we can provide a more efficient and versatile approach to enzyme kinetics.

Despite advancements in experimental enzymatic analysis, challenges in accurately determining kinetic parameters remain due to the complexity of biochemical systems, strict experimental conditions, and model limitations. Factors like pH and temperature add to the difficulty. Developing robust computational models is beneficial for predicting enzyme behavior and improving our understanding of enzymatic processes under various conditions, complementing traditional experimental methods. Advancing the evaluation of new machine learning methods [21] is crucial for analyzing large datasets, identifying trends [23], and predicting enzyme behavior. This progress enhances artificial enzyme discovery and optimization in biotechnology and medicine. This study focuses on understanding peroxidase activity, particularly heme-based peroxidases, using a GFET system to monitor reactions in two modes (see Fig. 1).

1. **Transconductance measurement** involves measuring the drain-source current ($I_{\textrm{ds}}$) while maintaining a constant drain-source voltage ($V_{\textrm{ds}}$) and varying the gate voltage ($V_{\textrm{g}}$). In this configuration, the enzyme is immobilized on the graphene surface, where enzyme-substrate interactions induce changes in the graphene’s electronic properties. By varying $V_{\textrm{g}}$ and recording $I_{\textrm{ds}}$, transfer characteristic curves can be generated, revealing the influence of enzymatic activity on the electronic environment of the graphene layer. This technique offers high sensitivity and real-time monitoring of enzymatic reactions, with Dirac voltage reflecting changes in local electronic properties due to enzymatic processes. Additionally, this method facilitates the investigation of peroxidase activity mechanisms, heme bleaching, and H$_2$O$_2$-induced suicide inactivation by analyzing shifts in the Dirac voltage in response to structural changes in the enzyme under varying H$_2$O$_2$ concentrations.
2. In **MM kinetics estimation** $I_{\textrm{ds}}$ is measured at constant $V_{\textrm{ds}}$ and $V_{\textrm{g}}$ to determine enzymatic parameters based on MM kinetics. The substrate concentration is varied while maintaining fixed $V_{\textrm{ds}}$ and $V_{\textrm{g}}$, and the corresponding $I_{\textrm{ds}}$ is recorded. The relationship between $I_{\textrm{ds}}$ and substrate concentration is analyzed to extract critical enzymatic parameters, including the Michaelis constant ($K_{\textrm{m}}$) and the maximum reaction rate ($V_{\textrm{max}}$). By fitting the MM equation, which characterizes the dependence of reaction rate on substrate concentration, to the experimental data, key insights into the enzyme’s catalytic efficiency and substrate affinity are obtained.

In this work, we concentrate on monitoring the enzymatic reactions using GFETs in the following aspects:

- The limitations of employing GFET systems for studying enzymatic reactions, particularly for determining MM constants, arise from the complexities of enzyme kinetics, the non-specific and sensitive response of GFETs, and the challenges in directly correlating Dirac voltage shifts with enzymatic reaction rates. These factors suggest that while GFETs offer powerful biosensing capabilities, they may necessitate additional calibration, alternative measurement strategies, or complementary techniques to accurately investigate complex enzymatic systems, such as heme-containing peroxidases. We explore these constraints within GFET systems and determine the MM constants for heme-containing peroxidase enzymes, such as HRP. This analysis will clarify why the traditional approach of linking Dirac voltage shifts to enzymatic reaction rates may not be effective in this context.
- In GFET-based biosensing, the direct electron transfer (DET) effect involves redox-active species in the environment engaging in direct electron exchange with the graphene surface. This interaction complicates the accurate interpretation of Dirac voltage shifts, a key parameter used to monitor enzymatic activity. The redox shift associated with the Faradaic current, which resembles a doping-like effect, is observed to be non-Nernstian and influenced by factors well-known in electrode kinetics, including electrode surface area, the standard potential of redox probes, and the scan rate of gate voltage modulation [26]. While this article does not explore the complex interactions between these environmental parameters and enzyme activity in detail, we recognize their significance. To provide context, we reference prior studies and recent findings that discuss structural changes in HRP and its heme group. However, fully elucidating the extent to which environmental redox species contribute to or interfere with these changes is beyond the scope of this discussion.
- We investigate the complex mechanisms governing enzymatic reactions in heme-containing peroxidase enzymes, with a particular emphasis on the impact of hydrogen peroxide. Specifically, we examine the phenomenon of suicide inactivation [4], a process in which hydrogen peroxide progressively inactivates the peroxidase enzyme, ultimately disrupting the entire reaction pathway. By analyzing this self-inactivation mechanism, we aim to elucidate the effects of hydrogen peroxide on the catalytic activity and stability of heme-containing peroxidases, offering valuable insights for both biochemical applications and the modeling of enzyme kinetics.
- We ensure that the changes in $I_{\textrm{ds}}$ can be precisely correlated with enzyme activity rates, providing a more reliable assessment of enzyme function in various biochemical contexts.
- We strive to enhance the accuracy and efficiency of analyzing enzymatic activity in GFETs. By using machine learning techniques, the methodology aims to overcome the limitations of traditional calculation methods, providing more precise estimates of MM constants and thereby improving the overall understanding and application of enzymatic kinetics in GFET-based biosensors.

## Materials and methods

### Materials

HRP (Type VI) was obtained from Sigma-Aldrich as a lyophilized powder and used without further purification. The specific activity of the enzyme, as reported by the manufacturer, is 250 units/mg, where one unit is defined as the amount of enzyme required to produce 1 mg of purpurogallin from pyrogallol within 20 s at pH 6 and 20 $^{\circ }\textrm{C}$. Hydrogen peroxide (30 wt%) was also procured from Sigma-Aldrich. All other reagents used in this study were of analytical grade and sourced from Sigma-Aldrich.

### Instruments

Atomic force microscopy (AFM) analysis was conducted using an Agilent 5500 instrument in AC tapping mode. SCOUT70 tips with an average radius of 15 nm were employed, with a scan resolution of 512$\times$512 pixels. Scans were performed under dry conditions, and the resulting images were analyzed using Gwyddion software to quantify particle density on the graphene surface. X-ray photoelectron spectroscopy (XPS) analysis was carried out using a Nexsa G2 instrument from Thermo Fisher. The primary objective of this analysis was to determine elemental concentrations and the chemical composition of the samples. For UV–Vis analysis, NanoDrop One/One Microvolume UV–Vis Spectrophotometers were employed.

The GFET S-20 chip, produced by Graphenea, is specifically engineered for enzymatic reaction analysis within a two-dimensional FET system. The chip comprises 12 graphene-based devices, each featuring encapsulated metal pads to prevent degradation and reduce leakage currents. Probe pads are strategically placed near the periphery for convenient access. A key feature of the GFET S-20 chip is the centrally located non-encapsulated gold (Au) electrode, which facilitates liquid gating without the need for an external gate electrode, thereby simplifying functionality and enhancing usability. The manufacturing process of the GFET S-20 chip involves several critical steps. It begins with the chemical vapor deposition (CVD) of graphene on copper foil, followed by the transfer of the graphene onto a Si/SiO$_2$ substrate. The graphene is then patterned using photolithography and O$_2$ plasma etching. Subsequent steps include gold metallization and Al$_2$O$_3$ passivation via atomic layer deposition, culminating in the dicing of the chip into individual devices. The individual graphene FETs on the chip exhibit carrier mobilities exceeding $1000\,\mathrm {cm^2V^{-1}s^{-1}}$ on average, with the Dirac point consistently below 1000 mV across 10 batches of devices. Quality control indicates an average yield of over 75% per wafer. In summary, the GFET S-20 chip delivers high-performance graphene devices with exceptional yield and functionality, making it a superior choice for enzymatic reaction analysis in two-dimensional FET systems.

A commercial readout system was employed for efficient and precise measurement of the electrical response of GFETs. Voltage signals for both the drain and gate were generated using a Keithley SMU device model 2612B. Prior to measurements, the graphene surface was cleaned of trace metal impurities. This was achieved by cycling the gate voltage between −500 and 500 mV in a 100 mM HCl solution until the Dirac point stabilized. At the beginning and end of each measurement series, the field effect was recorded in the same blank buffer to identify and account for any potential drifts introduced during the series. The same device configuration was utilized for electrochemical experiments. In three-electrode electrochemical measurements, a platinum wire was introduced as the counter electrode, while both graphene contacts served as the working electrode to ensure complete contact across the graphene channel. A commercial Ag/AgCl reference electrode was used for cyclic voltammetry.

For transconductance measurements in the FET system, the gate potential was scanned from 0 to 1000 mV at a rate of 20 mV/s, while the drain voltage was maintained at 20 mV. Measurements were performed using only a forward scan, with no reverse scans. To minimize the risk of cross-contamination, the solution was replaced for each test, and the chip surface was thoroughly washed with buffer three times before introducing a new solution. The test medium consisted of a 1 mM phosphate buffer solution containing 1 mM KCl as the supporting electrolyte. To ensure that no water electrolysis occurred within our applied voltage range, we tested the chip using cyclic voltammetry in the range of 0 mV to 1200 mV. No increase in current was observed in the blank solution, confirming the absence of electrolysis under these conditions (Fig. S1).

### HRP immobilization procedure

The immobilization of HRP on the graphene surface was conducted according to a standardized protocol. The GFET chip was submerged in a solution of 10 mg/mL of PBASE (1-pyrenebutanoic acid succinimidyl ester) dissolved in dimethylformamide (DMF) for 2 h. The pyrene groups in PBASE facilitated non-covalent functionalization through irreversible adsorption onto the intrinsically hydrophobic graphene surface, as described by Chae et al. [27]. Following immobilization, the chip underwent a series of washes with DMF and phosphate buffer. Next, a solution of 25 mg/mL HRP in phosphate buffer was applied to the graphene chip for 2 h. After this incubation, the chip was thoroughly rinsed and immersed in Tris buffer at pH 9 for 5 min to promote the hydrolysis of any unreacted PBASE, which could enhance the graphene surface’s sensitivity to pH changes. The chip was then rinsed three times with phosphate buffer at pH 7. The amount of HRP immobilized on the graphene was quantified by measuring the residual HRP concentration in the original solution after adsorption.

### Heme immobilization procedure

The immobilization of heme on the GFET was accomplished by immersing the GFET in a 5 mg/mL heme solution in dimethyl sulfoxide (DMSO) for 2 h. Following this incubation, the GFET was thoroughly rinsed with DMSO and phosphate buffer to remove any unbound material. The $\pi -\pi$ stacking interactions between the porphyrin ring of the heme molecule and the graphene surface are anticipated to result in a robust and efficient immobilization of heme on the graphene substrate, as reported by Xue et al. [28]. The quantity of heme adsorbed onto the graphene was quantified by calculating the difference between the initial concentration of heme in the solution and the residual concentration in the supernatant post-immobilization.

### GFET examination

In the investigation of the enzymatic mechanism following the optimization process, $V_{\text {ds}}$ was set to 20 mV, while $V_{\text {gs}}$ was scanned from 0 to +1000 mV. Ascorbic acid (AA) was chosen as a cosubstrate due to its ability to stabilize pH across a wide range during the reaction. AA, a well-known cosubstrate of H$_2$O$_2$ in peroxidase-catalyzed reactions, undergoes oxidation to form dehydroascorbic acid, a product significantly less acidic than its precursor. This pH shift results in a marked increase in pH, subsequently causing a significant shift in the Dirac voltage of the GFET towards a more positive value. This phenomenon is useful for studying the electrochemical properties of enzymatic reactions [29].

$$\begin{aligned} \text {Ascorbic Acid} + \text {H}_2\text {O}_2 \longrightarrow \text {dehydroascorbic acid} + \text {H}_2\text {O} \end{aligned}$$

To calculate the enzymatic parameters, 2,2’-azino-bis(3-et-hylbenzothiazoline-6-sulfonic acid) (ABTS) was employed. Enzyme activity parameters, as defined by MM kinetics, were determined by measuring $I_{ds}$ under fixed $V_{\text {ds}}$ and $V_g$ conditions. Specifically, $V_{\text {ds}}$ was held constant at 500 mV, while $V_{\text {gs}}$ was set to 500 mV for HRP and 100 mV for heme.

**Fig. 2:**
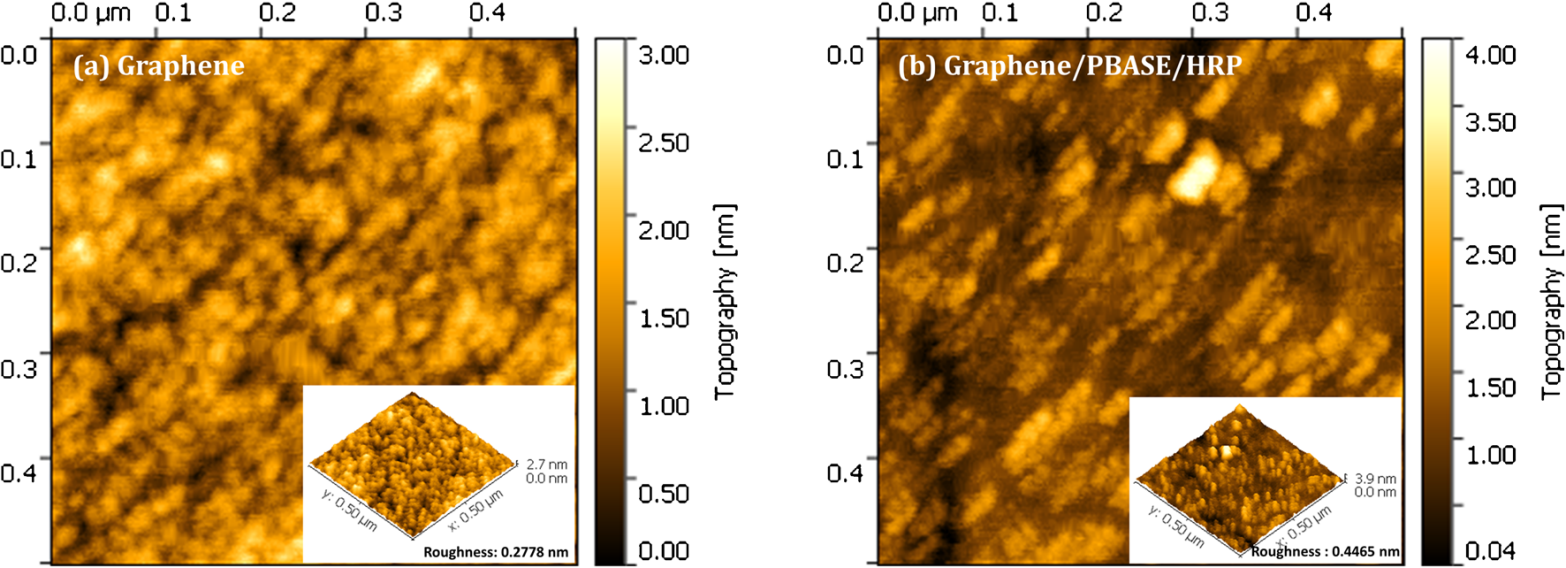
AFM images of graphene (**left**) and graphene immobilized with PBASE as a cross-linker (**right**)

## Multi-layer perceptron deep neural networks Bayesian inversion (MLP-DNN-BI) for enzyme reactions

The MM equation describes the rate of enzymatic reactions by relating the reaction rate to substrate concentration. In terms of the substrate concentration ($\text {S}$), enzyme concentration ($\text {E}$), enzyme-substrate complex ($\text {C}$), and product concentration ($\text {P}$), the reaction pathway can be represented as follows:

1 $$\begin{aligned} \text {E}+\text {S} \overset{k_2}{\underset{k_1}{\leftrightharpoons }\ }\text {C} \xrightarrow []{k_{\text {cat}}} \text {E}+\text {P}, \end{aligned}$$

where $k_{\text {cat}}$ is the catalytic rate constant, and the MM constant ($K_\textrm{M}$) is given by $K_\textrm{M}=\left( k_2+k_{\text {cat}}\right) /k_1$. In order to compute the MM parameters effectively, we use a mathematical modeling based on ordinary differential equations (see Section S1). Bayesian inversion for parameter estimation provides a probabilistic framework that combines prior knowledge with observed data to infer parameter distributions, offering a more comprehensive alternative to single-point estimates (see Section S2). This approach is applied to accurately estimate the parameters of the MM kinetics.

We use a designed MLP-DNN-BI algorithm for identifying MM parameters and predicting enzyme behavior. Using Bayesian inversion we estimate posterior densities $K_{\text {cat}}$ and $K_\textrm{M}$. The model employs unsupervised learning to predict MM parameters, linking enzyme reaction rates with features like substrate concentration, pH, temperature, and enzyme type. Trained models are saved for future predictions or fine-tuning (see Section S3). Figure S2 provides an overview of the algorithm, which combines deep learning (architecture depicted in Fig. S3) with Bayesian inversion to enhance predictive accuracy and effectively account for parameter estimation.

## Surface modification examination

### AFM

HRP was covalently attached to the graphene surface using PBASE, a well-established bifunctional compound containing both a pyrene group and a succinimidyl ester. The hydrophobic graphene thin film was noncovalently functionalized via the strong $\pi -\pi$ stacking interaction between the pyrene group of PBASE and the basal plane of the graphene. Simultaneously, the succinimidyl ester groups of PBASE formed stable amide bonds with the amine groups of HRP, ensuring covalent attachment. AFM was employed for topographic analysis to confirm the modification of the graphene surface with HRP. Figure 2(left) shows the characteristic structure of the deposited graphene sheet, which exhibits a root mean square roughness of 0.28±0.043 nm in its pristine state. Subsequent analysis using Gwyddion software revealed that the surface roughness of the HRP/PBASE/Graphene interface increased to 0.45±0.065 nm, as depicted in Fig. 2(right). These results confirm the successful immobilization of HRP enzyme molecules on the graphene surface via PBASE as a linker, consistent with the findings of Chae et al. [27].

### XPS

X-ray photoelectron spectroscopy (XPS) was utilized to confirm the functionalization of PBASE, HRP, and heme on the graphene surface. Figure S4 presents the C1s XPS spectra for (a) graphene, (b) PBASE-functionalized graphene, and (c) HRP/PBASE-functionalized graphene, along with the N1s XPS spectra for (d) PBASE/Graphene, (e) HRP/PBASE/Graphene, and (f) a comparison of the N1s peak intensities between (d) and (e). The deconvolution of the C1s spectrum for graphene revealed four types of carbon bonds: C-C (284.6 eV), C-O (286.7 eV), C=O (287.8 eV), and O-C=O (288.7 eV). In the C1s spectrum of PBASE-functionalized graphene (Fig. S4 b), a new peak emerged at 286.5 eV, corresponding to C-N bonds in PBASE molecules conjugated to the graphene sheets, alongside the presence of O-C=N bonding in the high-resolution deconvoluted C1s spectra centered at 289.7 eV. These observations confirm the effective binding of PBASE to graphene. Upon HRP incubation, the high-resolution C1s spectra exhibited increased peak intensities at C-C (285.0 eV), C-O/C-N (286.6 eV), and O-C=N (288.3 eV), which can be attributed to the amine and amide groups present in HRP [28]. The examination of the N1s region revealed the appearance of an additional N1s peak in PBASE-functionalized graphene compared to graphene, confirming successful N-doping following PBASE functionalization. The further increase in the N1s peak intensity after HRP immobilization indicates the presence of amide nitrogen atoms derived from the peptide moieties of HRP.

**Fig. 3:**
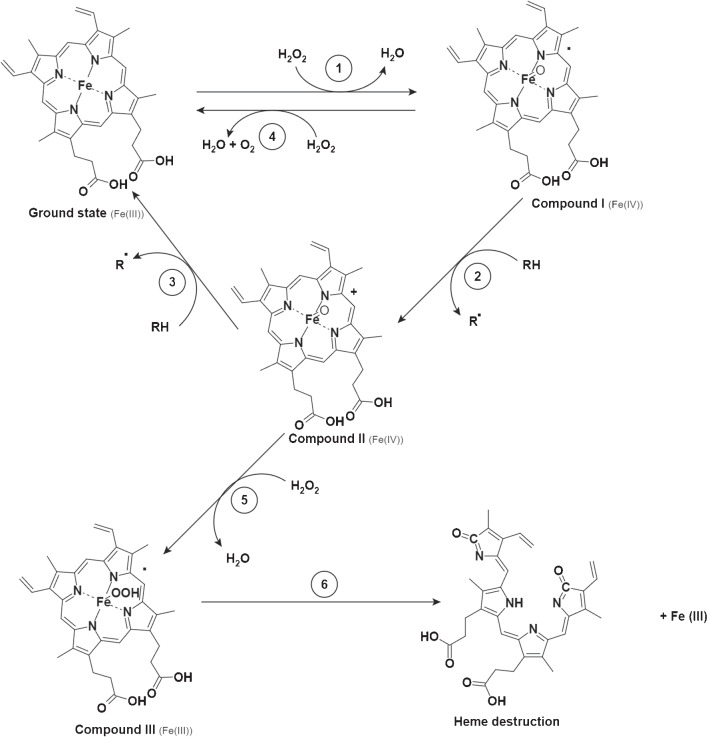
The key steps and reactions involved in the catalytic process of peroxidases

Figure S5 shows (a) the XPS survey spectrum, (b) the high-resolution C1s spectrum, and (c) the high-resolution N1s spectrum of Heme/Graphene. The XPS survey spectrum shows Fe2p signals, indicating the noncovalent functionalization of heme on the graphene surface. In comparison with the XPS spectra of the C1s core level for bare graphene, the deconvolution of the C1s spectrum for heme/Graphene reveals a new peak at 285.7 eV, corresponding to C-N bonds in heme/Graphene [30, 31]. Examination of the XPS N1s region further confirms successful immobilization of heme by the appearance of an additional N1s peak in heme/Graphene. The presence of N1s and Fe2p peaks, both originating from heme, conclusively verifies the successful noncovalent functionalization of graphene with heme [32].

### FET

To confirm the presence of PBASE on the graphene surface, $I_{\textrm{ds}}-V_{\textrm{g}}$ characteristics were measured before and after surface modification. Figure S4 a illustrates the $I_{\textrm{ds}}$ versus $V_{\textrm{g}}$ for bare graphene, following PBASE immobilization, and subsequent HRP immobilization on the graphene FET. The immobilization of PBASE resulted in a positive shift in the Dirac voltage, indicating an increase in electron density on the graphene surface. This shift was further observed after HRP immobilization, which introduced additional negative charges and caused a further shift in the Dirac voltage, as reported by [33]. Additionally, the $I_{\textrm{ds}}$ versus $V_{\textrm{g}}$ characteristics for heme immobilization on the graphene surface were analyzed. The data presented in Fig. S6 b show that heme immobilization also led to a positive shift in the Dirac voltage, consistent with an increase in electron density on the graphene surface.

## Results and discussions

### Examination of suicide inactivation effect

Heme-based peroxidases are prone to instability, particularly through a process known as suicide inactivation, where the enzyme is deactivated by its substrate, H$_2$O$_2$. This inactivation is most severe in the absence of reducing substrates, and its underlying mechanism has been partially understood. Extensive research on classical peroxidases has established a consensus catalytic network (Fig. 3). This begins with the formation of a sixth-coordination bond between hydrogen peroxide and the heme iron, producing compound I, a high-valent oxo-iron(IV) porphyrin-based $\pi$-free radical (pathway 1). In the presence of a two-electron reducing agent (e.g., an aromatic compound), compound II forms (pathway 2), which then oxidizes another substrate molecule, reverting to the resting-state iron(III) porphyrin (pathway 3). Without a reducing substrate, H$_2$O$_2$ addition leads to the formation of compound III (pathway 5). Further H$_2$O$_2$ exposure causes compound III to undergo bleaching and irreversible inactivation (pathway 6) [4].

Once compound III is formed, it may undergo various decomposition pathways. Given the proximity of the bound peroxy radical or compound III to the porphyrin ring, it is plausible that this reactive species could oxidize the porphyrin moiety, leading to the release of Fe(III) (pathway 6). However, the addition of excess reducing substrate can mitigate suicide inactivation by competing with H$_2$O$_2$ for compound II [4]. Electrochemical studies demonstrate that graphene electrodes effectively facilitate electron transfer with redox-active molecules in solution, resulting in faradaic currents. Unlike bulk semiconductor-based ISFETs, which include an insulating layer that impedes electron transport, graphene enables these interactions [34].

We aim to investigate the peroxidase behavior of HRP and the heme molecule using a GFET system. The standard method for assessing enzyme activity with GFETs typically involves monitoring the Dirac voltage shift resulting from pH changes induced by enzymatic reactions [27, 35, 36]. However, our observations suggest that the Dirac voltage shift in GFETs cannot be solely attributed to pH changes. We found that adding excess H$_2$O$_2$ leads to unexpected variations in the Dirac voltage shift. We attribute this complexity to two key factors: (1) DET within the GFET system influenced by redox-active materials, and (2) structural modifications induced by H$_2$O$_2$ on HRP and heme.

Redox-active molecules in the analyte solution can facilitate heterogeneous electron transfer with graphene, generating a faradaic current in the FET configuration and shifting the Dirac point. These shifts become significant when the faradaic current is substantial, often due to a large graphene surface area. This redox shift, driven by the faradaic current and resembling a doping effect, is non-Nernstian and depends on factors familiar in electrode kinetics, such as electrode area, the standard potential of the redox probes, and the scan rate of gate voltage modulation. The heme molecule is inherently redox-active, comprising an iron ion coordinated within a porphyrin ring. This iron ion can transition between various oxidation states, most commonly Fe(II) and Fe(III), enabling the heme molecule to participate in electron transfer reactions. This redox capability is essential for the function of many heme-containing proteins and enzymes, such as hemoglobin, myoglobin, cytochromes, and peroxidases like HRP. These processes are crucial for oxygen transport, electron transfer in cellular respiration, and catalytic reactions in peroxidases. While the interference of electron transfer in Dirac shifts due to electroactive materials adds complexity to enzymatic studies, it presents a valuable opportunity to investigate enzymatic mechanisms, including H$_2$O$_2$-induced suicide inactivation and heme bleaching.

**Fig. 4:**
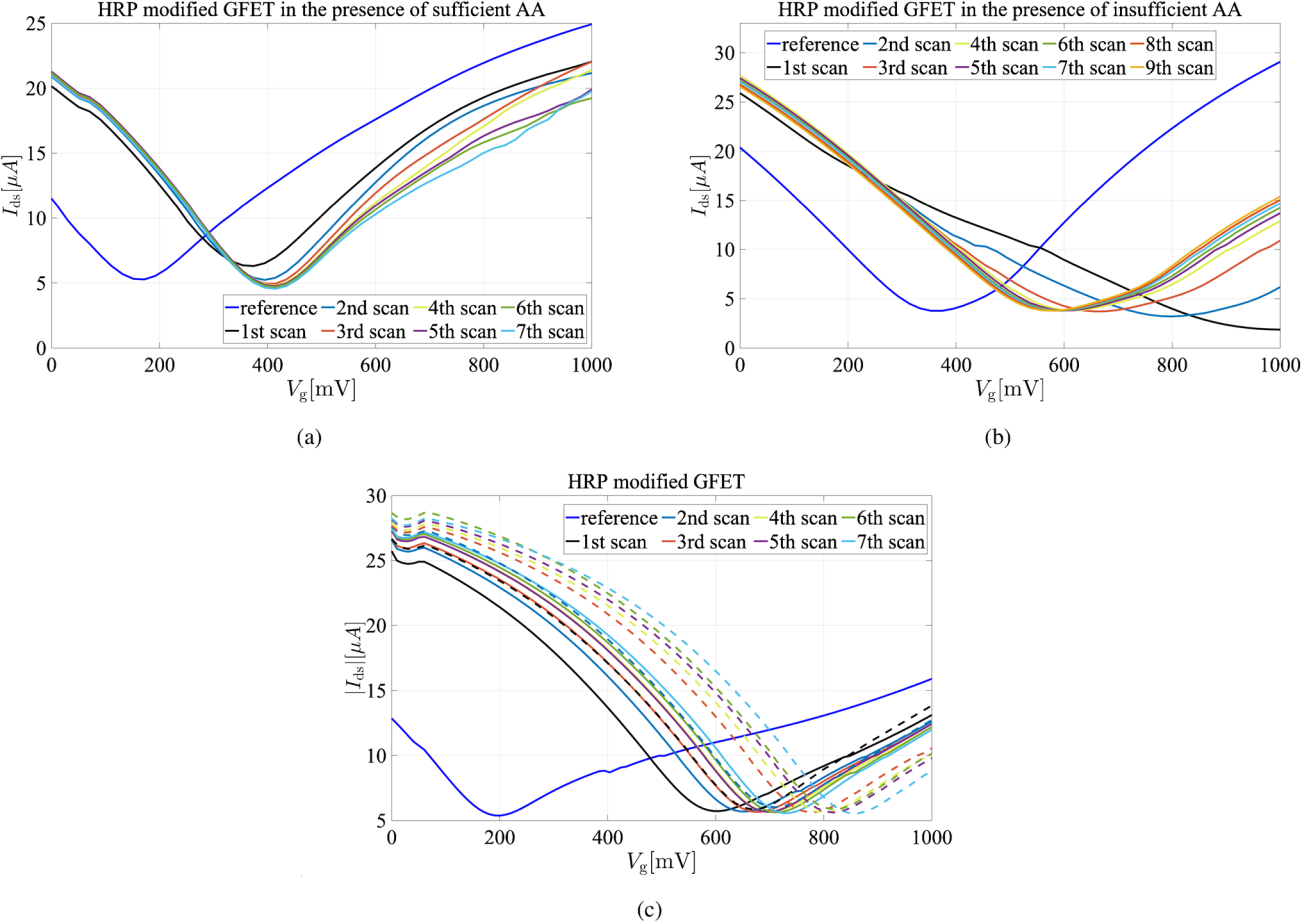
$I_{\textrm{ds}}$ versus $V_{\textrm{g}}$ obtained from curves of continuous scanning of the GFET at 5-second intervals at $V_{\textrm{g}}=20\,\textrm{mV}$ for **a** the HRP-modified GFET, in the presence of 50 $\mu$M H$_2$O$_2$ with cosubstrate concentrations of $500\,\mathrm {\mu M}$(sufficient), **b** $75\,\mathrm {\mu M}$ (insufficient) AA, and **c** heme- modified in the presence (solid lines) and absence (dashed lines) of $100\,\mathrm {{\mu } M}$ H$_2$O$_2$ in the absence of AA

Figure 4a and b show the $I_{\textrm{ds}}$ versus $V_{\textrm{g}}$ curves at a $V_{\textrm{ds}}$ of 20 mV for the HRP-modified GFET, with measurements conducted in the presence of 50 $\mu$M H$_2$O$_2$ and either $500\,\mathrm {\mu M}$ (sufficient) or $75\,\mathrm {\mu M}$ (insufficient) AA as a cosubstrate. The $V_{\textrm{g}}$ was scanned at 5-second intervals (*i*th scans), beginning with a *reference* scan. With sufficient cosubstrate, a gradual shift in the Dirac potential toward more positive voltages was observed. This shift is attributed to the consumption of AA during the enzymatic reaction and its conversion to less acidic products, resulting in an increase in pH and a corresponding positive shift in the Dirac voltage until the reaction reached a steady state. In the absence of adequate ascorbic acid, an initial significant increase in the Dirac voltage to a more positive value is observed. This is subsequently followed by a gradual decline of the Dirac voltage towards a more negative potential, eventually stabilizing at a steady-state value.

Figure S7 presents the changes in Dirac voltage as a function of time under two distinct conditions: the presence of sufficient and insufficient amounts of AA. These measurements provide a direct comparison of the system’s response to varying AA concentrations. To ensure the reliability of the results, we also monitored the Dirac voltage of HRP-modified graphene in the absence of both H$_2$O$_2$ and AA. This control experiment was conducted to rule out any potential drift in the voltage over time, thereby confirming that the observed changes are solely attributable to the interaction between the analytes and the modified graphene surface.

In [2], HRP states under varying H$_2$O$_2$ concentrations were examined using UV–Vis spectroscopy. At low H$_2$O$_2$ levels, HRP mainly transitions between its ground state and compound I. The UV–Vis spectra show that initial H$_2$O$_2$ additions shift the Soret band from 403 nm (ground state) to 420 nm (compound II), with additional bands at 527 and 555 nm, indicating reversible transformations between these states. However, compound I formation was minimal, as evidenced by the absence of its characteristic bands. At higher H$_2$O$_2$ concentrations, HRP advances to the compound III state, marked by a Soret band shift to 417 nm, along with bands at 544 and 580 nm. Over time, the enzyme degrades into an inactive state, indicated by a reduction in the Soret band and the emergence of a 670 nm band, corresponding to biliverdin formation. This UV–Vis data from the Contreras group demonstrates that HRP’s state and stability are strongly influenced by H$_2$O$_2$ levels, with higher concentrations accelerating enzyme degradation and inactive species formation [2]. HRP is a heme-containing enzyme with an Fe(III) ion at its core, essential for its catalytic activity. Under excessive H$_2$O$_2$ exposure, HRP undergoes oxidative stress, leading to heme degradation and the release of free Fe(III) ions. This occurs as H$_2$O$_2$ reacts with the heme group, forming reactive intermediates such as compound III (Fe(III)-O$_2$), which can further damage the porphyrin ring and cause Fe(III) dissociation from the enzyme. The release of free Fe(III) ions into the solution significantly affects the electronic properties of the graphene surface. As redox-active species, Fe(III) ions can interact with graphene, leading to charge transfer that results in a p-doping effect, increasing positive charge density and shifting the Dirac voltage to a more positive value. This shift is initially pronounced due to the large release of Fe(III) ions following HRP degradation. However, as the system stabilizes and the Fe(III) concentration near the graphene surface equilibrates or diffuses away, the Dirac voltage may partially return toward its original value.

In the absence of sufficient AA, free Fe(III) ions remain in their oxidized state, sustaining their impact on graphene and causing a sharp positive Dirac voltage shift. AA typically moderates this impact by stabilizing the graphene’s electronic properties. With adequate AA, the Dirac voltage shift is more controlled and gradual, as AA prevents excessive Fe(III) release at the graphene surface, leading to a slower, steadier shift in the Dirac point as AA is consumed. Maintaining sufficient reducing substrate is thus crucial for preserving HRP’s structural integrity and function during reactions involving H$_2$O$_2$ and potential scanning. Insufficient co-substrate leads to excessive heme iron oxidation in HRP, causing structural changes, reduced enzymatic activity, and influencing Dirac voltage in GFETs through DET.

We also analyzed the Soret band of HRP under three conditions (Fig. S8): 100 $\mu$M HRP in phosphate buffer, HRP in the presence of 50 $\mu$M H$_2$O$_2$ and 75 $\mu$M AA (insufficient reducing agent), and HRP in the presence of 50 $\mu$M H$_2$O$_2$ and 500 $\mu$M AA (sufficient reducing agent). In the case of free HRP, the Soret band was observed at 403 nm. However, with insufficient AA, the Soret band shifted to 417 nm, indicating the formation of Compound III. Interestingly, when sufficient AA was present, the Soret band returned to 403 nm, demonstrating that the enzyme, in the presence of an adequate reducing agent, follows pathway 3 and fully recovers its original state. These UV–Vis results are in complete agreement with our observations from the GFET system, further validating the proposed mechanism.

To investigate DET of the heme molecule on the GFET system, we employed a distinct approach. After immobilizing heme on the graphene surface, the GFET was continuously scanned at 5-second intervals, both with and without 1 mM H$_2$O$_2$ at pH 4 (Fig. 4c). In both scenarios, a positive shift in the Dirac voltage was observed, attributed to DET from the iron within the heme. Notably, when scanning ceased, the Dirac voltage returned to its initial value. However, in the presence of H$_2$O$_2$, the positive shift was smaller, and even after washing the surface and repeating the test without H$_2$O$_2$, the Dirac voltage shift did not increase, suggesting H$_2$O$_2$ may have degraded some heme molecules on the surface. To provide greater clarity, we illustrated the variation in Dirac voltage over time in the absence and presence of H$_2$O$_2$, as shown in Fig. S7 b. Additionally, the change in Dirac voltage for bare graphene was measured in a blank buffer to confirm the absence of any drift in voltage, ensuring the reliability of the observed results. This positive Dirac voltage shift mirrors the cyclic voltammetry behavior observed in heme-graphene composites, indicative of a single-electron transfer process involving the iron core of the heme in the heme$_\text {ox}$/heme$_\text {red}$ pair [30].

Excess H$_2$O$_2$ induces irreversible structural changes in the heme protein, potentially leading to iron release and conversion of Fe(II) to biliverdin. This disruption impacts heme-graphene interactions, altering the electronic properties of the graphene surface and compromising the stability and functionality of heme-immobilized GFETs, which affects their performance in sensing applications. We used our electrochemical setup to gain deeper insights into this phenomenon, as depicted in Fig. S9. The device was tested under three distinct conditions: unmodified GFET in phosphate buffer at pH 4 (red line), heme-modified GFET in the absence of H$_2$O$_2$ (green line), and heme-modified GFET in the presence of 100 $\mu$M H$_2$O$_2$ (blue line). All measurements were conducted at a scan rate of 50 mV/s. In the absence of H$_2$O$_2$, the CV profile exhibited well-defined anodic and cathodic peaks characteristic of the redox activity of the heme molecule. However, these peaks were significantly suppressed in the presence of H$_2$O$_2$, indicating a marked reduction in redox-active species. This suppression is attributed to the degradation of the heme molecule by H$_2$O$_2$. The CV data strongly align with the results from GFET analysis, confirming the impact of H$_2$O$_2$ on the redox behavior of the heme-modified graphene system.

To accurately study HRP and heme enzyme parameters, an adequate supply of co-substrate is crucial to prevent irreversible inactivation, including heme bleaching. During enzymatic studies, operating within a gate potential that minimizes unwanted redox reactions and DET [26] is essential. Our findings indicate that maintaining a constant positive voltage from 100 up to +1000 mV ensures minimal DET via Fe(III) conversion, preserving the integrity of heme-based sensors and enabling accurate enzyme activity measurements.

**Fig. 5:**
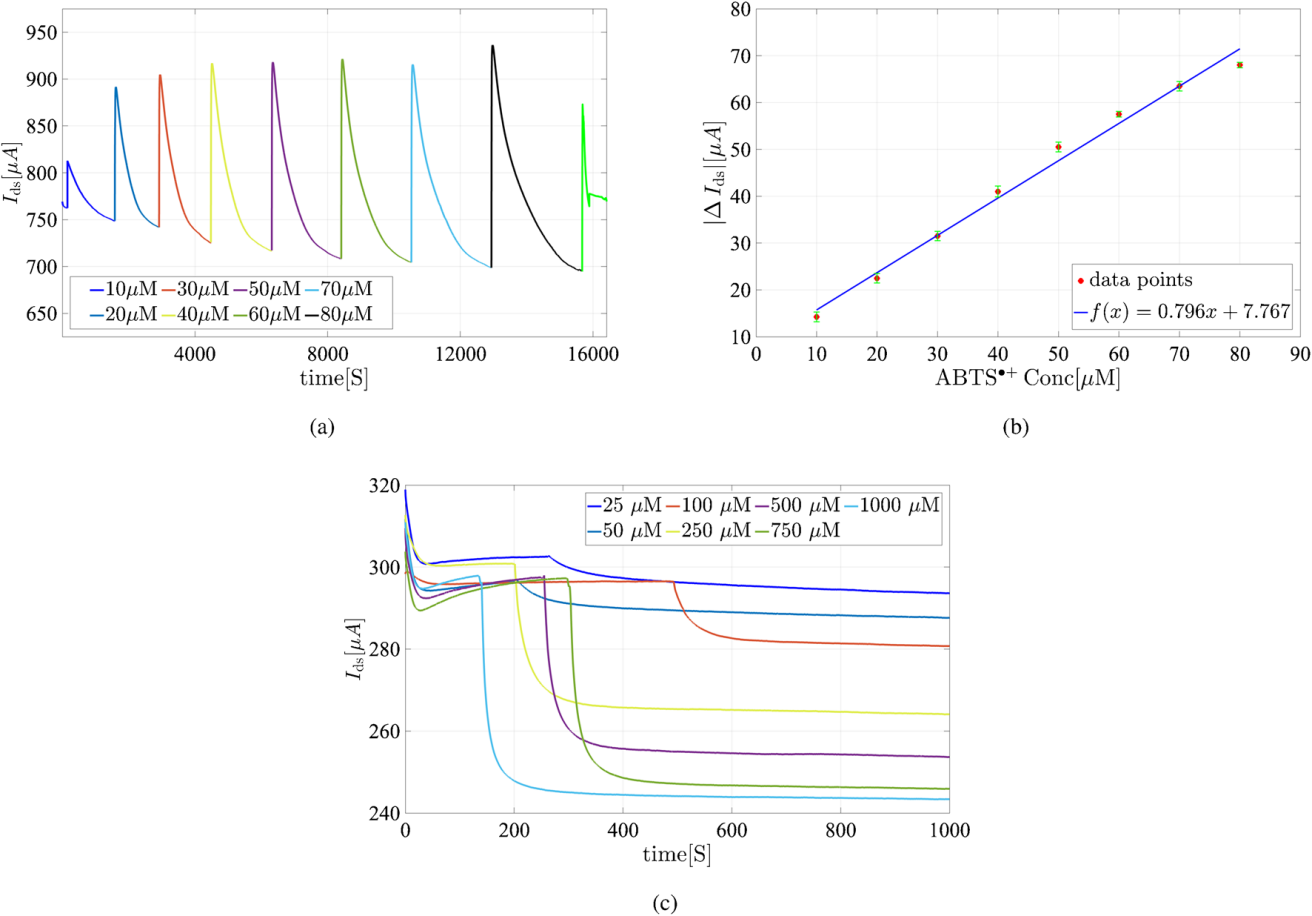
**a** The real-time response of the HRP-modified GFET ($I_{\textrm{ds}}$ vs. time) monitored as successive additions of $\text {ABTS}^{\bullet +}$ added to a 1 mM phosphate buffer containing 1 mM KCl (pH 7). $V_{\textrm{g}}$ and $V_{\textrm{ds}}$ were both maintained at a constant 500 mV. **b** Calibration curve derived from the real-time data. The results are presented with error bars to indicate the deviations from the expected values. **c** Real-time response of the HRP-modified GFET for various ABTS concentration in the presence of a sufficient amount of H$_2$O$_2$ (2.5 mM) in 1 mM phosphate contains 1 mM KCl (pH= 7). The applied $V_{\textrm{g}}$ and $V_{\textrm{ds}}$ were both set to 500 mV and held constant

### Enzymatic activity measurement

GFETs have highly sensitive surfaces capable of detecting subtle changes, but translating FET-based nanosensors into practical biomedical applications remains challenging, especially with carbon-based materials like graphene as the FET channel. These materials exhibit strong interactions with biomolecules through $\pi -\pi$ stacking, leading to significant nonspecific adsorption and false signals, which can overwhelm nanoscale FETs [37]. In liquid-gated FET setups, a specific gate voltage is applied, either constant or swept over a range, creating a potentiostatic situation. If the graphene channel undergoes electron transfer and redox-active species are present, an additional electrochemical current may flow, depending on the formal potential of the redox species and the gate voltage range [34] To prevent this, our detection method tracks the product current over time during the enzymatic reaction, maintaining constant gate and drain voltages. This approach minimizes the effect of electron transfer from redox materials within HRP or heme, ensuring accurate enzyme activity measurements. To address this issue, our platform detects enzyme activity by tracking the product current over time during the enzymatic reaction, while maintaining constant $V_{\textrm{g}}$ and $V_{\textrm{ds}}$ to minimize interference from electron transfer within HRP or heme. The enzymatic reaction is illustrated as follows, with ABTS selected as the cosubstrate:

$$\begin{aligned} { \text {H}_2\text {O}_2+2\,\text {ABTS}_{\text {reduced}}\longrightarrow \text {ABTS}^{\bullet +}_{\text {oxidized}}+2\, \text {H}_2\text {O}} \end{aligned}$$

**Fig. 6:**
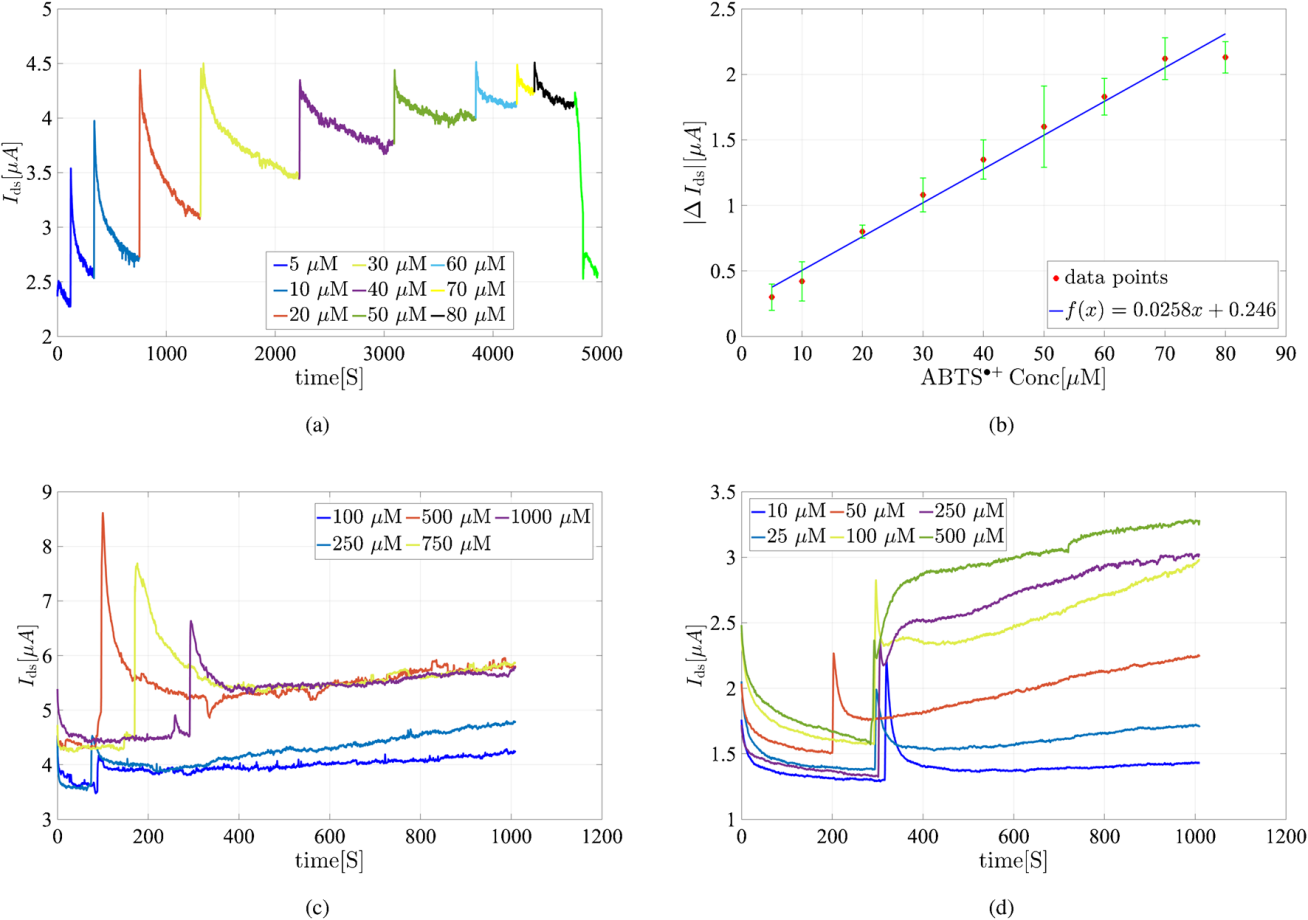
**a** Real-time response of the heme-modified GFET ($I_{\textrm{ds}}$ vs. time) upon incremental addition of $\text {ABTS}^{\bullet +}$ to a 1 mM phosphate buffer containing 1 mM KCl (pH = 4). **b** Calibration curve derived from the real-time response data. **c** Response of the heme-modified GFET in the presence of a various ABTS concentration and a fixed concentration of H$_2$O$_2$ ($2.5\,\textrm{mM}$) in a 1 mM phosphate buffer containing 1 mM KCl (pH= 4). **d** Response of the heme-modified GFET to different concentrations of H$_2$O$_2$ in the presence of a fixed amount of ABTS ($5\,\textrm{mM}$) in a 1 mM phosphate buffer containing 1 mM KCl (pH= 4). For all measurements, we maintain $V_{\textrm{g}}= 100\,\textrm{mV}$ and $V_{\textrm{ds}}= 500\,\textrm{mV}$

To optimize sensitivity, it is essential to measure the signal at different constant $V_{\textrm{g}}$ to identify the voltage at which the system is most sensitive to $\text {ABTS}^{\bullet +}$ and least sensitive to ABTS or H$_2$O$_2$. H$_2$O$_2$ is known to decompose catalytically, forming ${\text {HO}^{\bullet }}$ radicals [38], and ABTS addition causes subtle current changes in the GFET reservoir. For HRP, the optimal gate voltage was determined by measuring $I_{\textrm{ds}}$ versus $V_{\textrm{g}}$ at constant concentrations of $100\,\mathrm {\mu M}$ for H$_2$O$_2$, ABTS, and $\text {ABTS}^{\bullet +}$. Based on the data in Fig. S10 a, $V_{\textrm{g}}$ = 500 mV was selected as the optimal voltage, yielding significantly higher current for $\text {ABTS}^{\bullet +}$ compared to ABTS and H$_2$O$_2$. Similarly, for the heme molecule, $V_{\textrm{g}}$ = 100 mV was determined as optimal, as it produced a notably higher current for $\text {ABTS}^{\bullet +}$ than for ABTS and H$_2$O$_2$ (Fig. S10 b). To determine enzymatic parameters, a calibration curve for $\text {ABTS}^{\bullet +}$ was established using the GFET system’s response to varying $\text {ABTS}^{\bullet +}$ concentrations. $\text {ABTS}^{\bullet +}$ was generated via the reaction between ABTS and thiosulfate, a method commonly employed in antioxidant capacity assays like the Trolox Equivalent Antioxidant Capacity (TEAC) assay. GFET current measurements were taken at different $\text {ABTS}^{\bullet +}$ concentrations for both HRP and heme. Figure 5b shows the calibration curve of the absolute current variation $(|\Delta I_{\text {ds}}|)$ for $\text {ABTS}^{\bullet +}$ in the HRP-modified GFET, demonstrating a linear range from $10\,\mathrm {\mu M}$ to $70\,\mathrm {\mu M}$, with each concentration measured three times. For enzymatic studies, ABTS was added to the GFET reservoir in concentrations of $25\,\mathrm {\mu M}$, $50\,\mathrm {\mu M}$, $100\,\mathrm {\mu M}$, $250\,\mathrm {\mu M}$, $500\,\mathrm {\mu M}$, $750\,\mathrm {\mu M}$, and $1000\,\mathrm {\mu M}$ in the presence of $10\,\mathrm {m\,M}$ H$_2$O$_2$ at an optimized pH of 7. Figure 5c shows the $I_{\textrm{ds}}$ versus *time* plot for the enzymatic analysis of HRP with ABTS as the substrate.

The same measurement procedure was applied to the heme-modified GFET. As shown in Fig. 6a, the $I_{\textrm{ds}}$ versus time profile was recorded after adding $\text {ABTS}^{\bullet +}$ to the heme-modified GFET solution at an optimized pH of 4. Upon the addition of $\text {ABTS}^{\bullet +}$, the $I_{\textrm{ds}}$ current increased significantly, following a linear trend up to an $\text {ABTS}^{\bullet +}$ concentration of $80\,\mathrm {\mu M}$. Notably, after washing the system, the $I_{\textrm{ds}}$ current returned to baseline. Measurements were repeated three times, and a calibration curve of $(|\Delta I_{\text {ds}}|)$ versus $\text {ABTS}^{\bullet +}$, concentration was established (Fig. 6b). The peroxide activity of heme was subsequently evaluated with both ABTS and H$_2$O$_2$. Figure 6c shows the enzymatic analysis of heme-modified graphene with various ABTS concentrations in the presence of 2.5 mM H$_2$O$_2$. The $I_{\textrm{ds}}$ current increased with $\text {ABTS}^{\bullet +}$ production until plateauing at $750\,\mathrm {\mu M}$ ABTS. The same procedure was used to examine the heme affinity for H$_2$O$_2$ in the presence of a sufficient amount of ABTS (Fig. 6d).

Based on the GFET surface dimensions and molecular concentrations determined experimentally for immobilized HRP and heme, it was estimated that approximately $1.08 \times 10^9$ HRP molecules and $2.7 \times 10^{10}$ heme molecules were immobilized on the $90 \, \mu \text {m} \times 90 \, \mu \text {m}$ graphene surface. The notably higher density of heme molecules is attributed to their smaller molecular size compared to HRP. However, the HRP-immobilized GFET exhibited greater sensitivity to $\text {ABTS}^{\bullet +}$ concentration, likely due to its enhanced capability to trap and transfer charge to the graphene layer, thereby improving the sensor’s sensitivity towards $\text {ABTS}^{\bullet +}$.

While the heme-immobilized GFET relies on the direct interaction of heme molecules with the graphene surface, the HRP-immobilized GFET benefits from the enzyme’s structural properties that facilitate efficient charge transfer. The differences in the observed current signal change upon $\text {ABTS}^{\bullet +}$ addition can be attributed to graphene’s bipolar characteristics and the relative position of the applied potential with respect to the Dirac point. These findings underscore the versatility and adaptability of graphene-based FETs for studying and optimizing different catalytic systems. By leveraging the unique properties of immobilized molecules, this work provides valuable insights into the development of highly sensitive and selective biosensors, paving the way for advancements in bioelectronics and enzymatic catalysis.

### Machine learning for enzymatic reactions

We utilize our computational framework to determine key enzymatic parameters, including $K_{\textrm{m}}$ and $K_{\text {cat}}$. The architecture of the fully connected deep neural networks is shown in Fig. S3. The input features include critical variables such as enzyme components (HRP and heme), temperature, substrate concentration, and pH values, which collectively define the biochemical environment of the enzymatic reactions. The output layer provides predictions for the MM parameters, $K_{\textrm{m}}$ (the substrate affinity constant) and $K_\text {cat}$ (the catalytic turnover rate), which are essential for understanding the enzymatic kinetics.

The hidden layers consist of multiple fully connected neurons that capture and model complex nonlinear relationships between the inputs and outputs, with activation functions and regularization techniques applied to enhance learning and prevent overfitting. Activation functions were selected as follows: ReLU for the hidden layers, to ensure non-linearity and efficient training, and a Sigmoid activation function for the output layer, to appropriately map the outputs to the desired range. The Adam optimizer was used to minimize the loss function due to its adaptive learning capabilities and fast convergence. This configuration was chosen based on extensive parameter tuning and cross-validation, ensuring an optimal balance between model complexity, accuracy, and computational efficiency.

The experimental data serving as input features for the DNN includes the product formation rate measured over time across a range of environmental and chemical conditions. Specifically, data was collected at two pH values (4 and 7) and temperatures ranging from 18 to 25$^\circ$C. Substrate concentrations varied between 25 and 1000 $\mu$M, with substrates including H$_2$O$_2$ and ABTS, tested for two enzyme components (HRP and Heme). As an illustrative example, Fig. S11 presents the product formation rate of HRP at varying substrate concentrations, measured at a pH of 7 and a temperature of 22$^\circ$C. The machine learning model was trained to predict $K_\textrm{M}$ and $K_{\text {cat}}$, under diverse environmental conditions (e.g., varying temperatures) and chemical scenarios (e.g., different substrate concentrations). Approximately 100 experimental cases were utilized to train the DNN algorithm, with the dataset partitioned into 80% for training and 20% for testing, ensuring robust performance evaluation and generalization of the model.

Starting with the GFET response, we map the current to product concentration. We then apply MLP-DNN-BI to calculate the MM parameters (see Section S3). Then, Bayesian inversion is used to identify the posterior density of the MM parameters. For example, the posterior distribution for the HRP production rate (depicted in Fig. S11) is shown in Fig. S12. Our trained model allows for parameter computation under various environmental (temperature) and chemical (pH value) conditions. The regression plot (Fig. S13) compares Bayesian inversion data with our neural network predictions, demonstrating the model’s predictive accuracy. Additionally, Fig. S13 highlights RMSE variations over training epochs, underscoring the model’s efficiency and accuracy. Table S1 provides a comparison of the estimated MM parameters from various references alongside our findings.

## Conclusions

This study investigated the application of GFETs as a sophisticated technique for biochemical analysis, focusing on the peroxidase activity of HRP and heme molecules. The results demonstrated that GFETs exhibited exceptional sensitivity and specificity, making them highly effective for detecting minute variations in enzyme activity—an essential factor for precise biochemical evaluations. By using the transconductance capabilities of GFETs, significant enzymatic processes such as HRP suicide inactivation and heme bleaching were examined under controlled conditions, allowing for precise quantification of peroxidase activity with minimal interference. Additionally, a developed DNN parameter estimation technique is applied to determine MM enzymatic parameters. The integration of GFETs with unsupervised learning produced promising results in enzyme analysis, highlighting the potential of this combined approach to enhance the precision and reliability of biochemical measurements. In conclusion, this work validated the effectiveness of GFETs for enzyme analysis and established a foundation for future research that combines GFET technology with DNN. This integration holds promise for advancing our understanding of enzyme kinetics and other complex biochemical phenomena, offering new opportunities for exploration and potentially transforming biochemical investigations.

## Supplementary Information

Below is the link to the electronic supplementary material.

Supplementary file 1 (pdf 3155 KB)

## Data Availability

No datasets were generated or analyzed during the current study.
