# Supplementary material for: Graphene-based FETs for advanced biocatalytic profiling: investigating heme peroxidase activity with machine learning insights
Source: Mikrochim Acta. 2025 Mar 3;192(3):199. doi: 10.1007/s00604-025-06955-y (PMC11876242; doi:10.1007/s00604-025-06955-y)
Supplement: Supplementary file 1 — (pdf 3155 KB) [file 604_2025_6955_MOESM1_ESM.pdf]

Supplementary Material

Graphene-based FETs for advanced biocatalytic profiling: Investigating heme peroxidase activity with machine learning insights

Samaneh Mirsian [a], Wolfgang Hilber [a], Ehsan Khodadadian [e], Maryam Parvizi [d,e], Amirreza Khodadadian [c,e], Seyyed Mehdi Khoshfetrat [b], Clemens Heitzinger [e], Bernhard Jakobyb [a]

[a] Institute of Microelectronics and Microsensors, Johannes Kepler University, Austria

[b] Faculty of Basic Science, Ayatollah Boroujerdi University, Boroujerd, Iran

[c] School of Computer Science and Mathematics, Keele University, UK

[d] School of Mathematics, University of Birmingham, UK

[e] Machine Learning Unit, TU Wien, Vienna, Austria

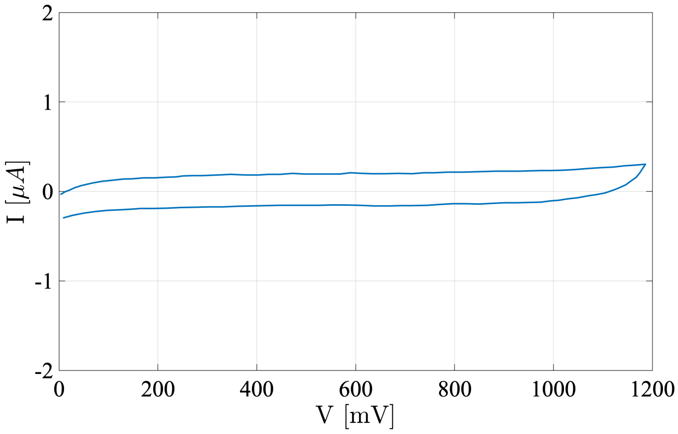

Figure S1: Three electrode CV for unmodified GFET in buffer device performance at a scan rate of 50 mV/s.

S1. Quasi-steady-state approximation based on ODEs

We apply a mathematical approach to simplify enzyme kinetics, especially under certain conditions These approximations help reduce the complexity of the differential equations governing enzyme kinetics. By differentiating (1) with respect to time, we obtain:

$$\frac{d\mathrm{S}}{dt} = -k_1, \mathrm{SE} + k_2, \mathrm{C},$$
$$\frac{d\mathrm{C}}{dt} = k_1, \mathrm{SE} - k_2, \mathrm{C} - k_{\mathrm{cat}}, \mathrm{C},$$
$$\frac{d\mathrm{P}}{dt} = k_{\mathrm{cat}}, \mathrm{C}.$$

We denote T_1 as the total enzyme concentration ($T_1 = \mathrm{C}+\mathrm{E}$) and T_2 as the total substrate and product concentration ($T_2 = \mathrm{S}+\mathrm{C}+\mathrm{P}$). Total Quasi-Steady-State Approximation (TQSSA) can be obtained by considering total enzyme concentration rather than free enzyme concentration, making it more accurate when the enzyme concentration is comparable to the

substrate concentration. TQSSA assumes equilibrium for the total enzyme-substrate complex and uses total substrate $T_3 = S + C$ instead of S [1]. Following [2], TQSSA is given by:

$$\frac{d\mathrm{P}}{dt} = k_{\mathrm{cat}} \frac{\mathrm{T}_1 + K_m + \mathrm{T}_2 - \mathrm{P} - \sqrt{(\mathrm{T}_1 + K_m + \mathrm{T}_2 - \mathrm{P})^2 - 4\mathrm{T}_1(\mathrm{T}_2 - \mathrm{P})}}{2}. \tag{2}$$

S2. Parameter estimation based on Bayesian inversion

Bayesian inversion is a statistical approach for estimating model parameters or states by integrating prior knowledge with observed data through Bayes' theorem. This technique is widely used across various domains, including geophysics, environmental science, engineering, and economics, to improve model predictions and quantify uncertainties. In particular, Bayesian inversion, combined with Markov Chain Monte Carlo (MCMC) methods, is highly effective for parameter estimation in complex, high-dimensional models. MCMC methods approximate the posterior distribution of model parameters by generating samples from it, providing the flexibility to handle non-Gaussian and nonlinear problems. Consider the following probabilistic model:

$$\mathcal{M} = \mathcal{Q}(x, \theta) + \varepsilon, \tag{3}$$

where $\mathcal{M}$ represents the n-dimensional measured data (in this context, enzyme production), and $\mathcal{Q}$ denotes the computational model (here, the MM equation, as in (S1) and (2)) influenced by MM parameters θ within the random field Θ. The observation error ε is assumed to be a Gaussian independent and identically distributed (iid) error, $\varepsilon \sim \mathcal{N}(0, \sigma^2 I)$, with variance σ^2. Given the measured data $\mathcal{M} = \mathtt{obs}$, the conditional density is defined as

$$\pi(\mathtt{obs}) = \int_{\mathbb{R}^n} \pi(\mathtt{obs}|\theta)\pi_0(\theta), \mathrm{d}\theta. \tag{4}$$

The posterior density is the central outcome of this process, reflecting the updated beliefs about the parameters after accounting for the data. It combines both the prior distribution and the likelihood, thereby balancing prior knowledge with new evidence. In parameter estimation, given a specific observation m, we aim to estimate the posterior distribution $\pi(\theta|m)$:

$$\pi(\theta|m) = \frac{\pi(m|\theta)\pi_0(\theta)}{\pi(m)}, \tag{5}$$

where $\pi_0(\theta)$ denotes the prior information (prior density). Using the statistical model (3), the likelihood function can be estimated as

$$\pi(\mathcal{M}|\theta) = L(\theta, \sigma^2|\mathcal{M}) = \frac{1}{(2\pi\sigma^2)^{n/2}} \exp\left(-\frac{\mathcal{EEM}}{2\sigma^2}\right), \tag{6}$$

where

$$\mathcal{EEM} = \sum_{j=1}^{n} \left[\mathcal{M}_j - \mathcal{Q}_j(x, \theta)\right]^2, \tag{7}$$

is the sum of squared errors. Different MCMC methods can be used to estimate the posterior densities, including Metropolis-Hastings, adaptive Metropolis, delayed rejection, delayed Rejection Adaptive Metropolis (DRAM), MCMC with Ensemble-Kalman Filter (EnKF-MCMC), etc. [3].

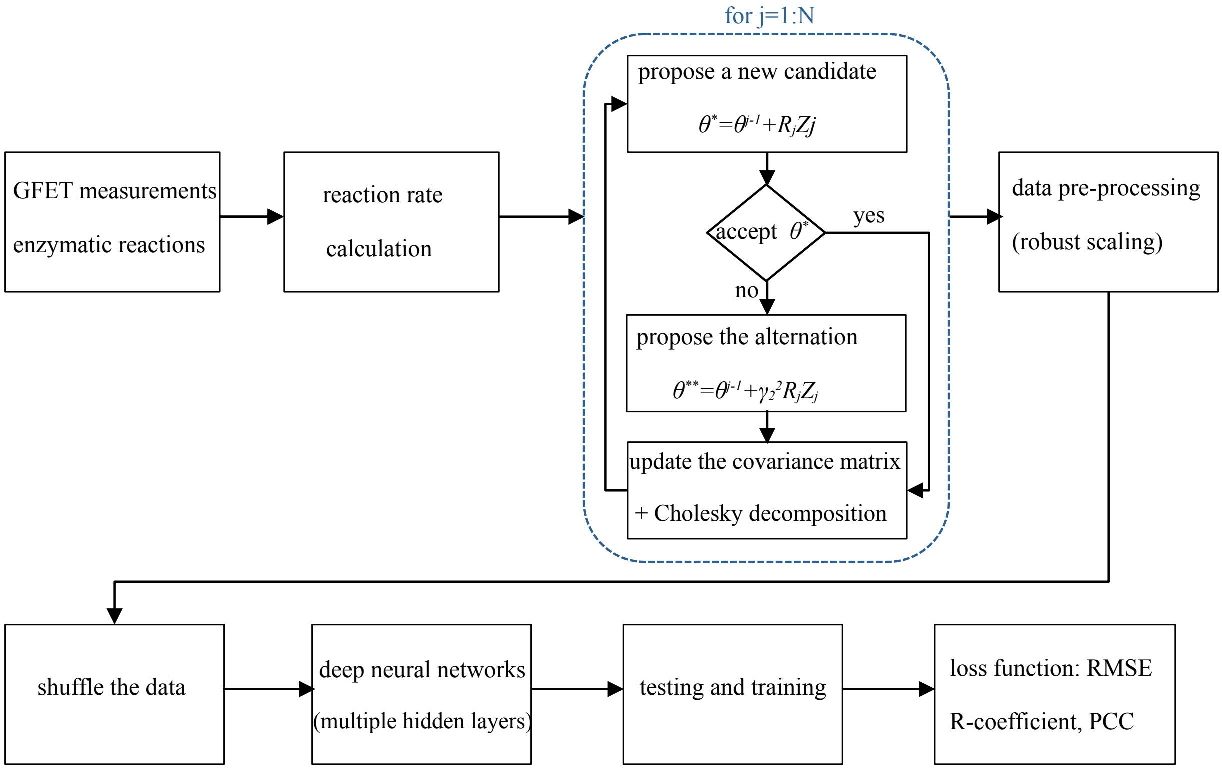

Figure S2: The structure of the MLP-DNN-BI model used to estimate the MM parameters based on the current measurement in GFET. The shown Bayesian inversion (given in the blue box) is DRAM. The DNN architecture is shown in Figure S3.

S3. Deep neural networks Bayesian inversion model

We introduce a new designed multi-layer perceptron deep learning Bayesian inversion algorithm to identify the MM parameters and train the machine to predict enzyme behavior. The algorithm is shown in Figure S2. The system integrates deep learning with Bayesian inversion to improve prediction accuracy and handle uncertainty in parameter estimation. The algorithm details are as follows.

1. For enzymatic analysis, H_2O_2 or $ABTS^{\bullet+}$ was added to the GFET reservoir. The current, converted to $ABTS^{\bullet+}$ concentration, was used to determine $V_{\max}$.

2. A calibration curve for $ABTS^{\bullet+}$ versus I_{ds} was plotted, mapping current (in μA) to product concentration (in μM) to calculate reaction rates. The details of the calibration curve technique for the data conversion are given in [4], Chapter 4.

3. Experimental rates were used to solve TQSSA for MM parameters estimation via MCMC, as described in [3].

4. Robust scaling normalized the data by removing the median and scaling according to the interquartile range (IQR), improving training stability.

5. Data shuffling was applied to prevent overfitting and enhance model generalization.

6. A deep neural network with ReLU and sigmoid activations was designed to capture complex relationships.

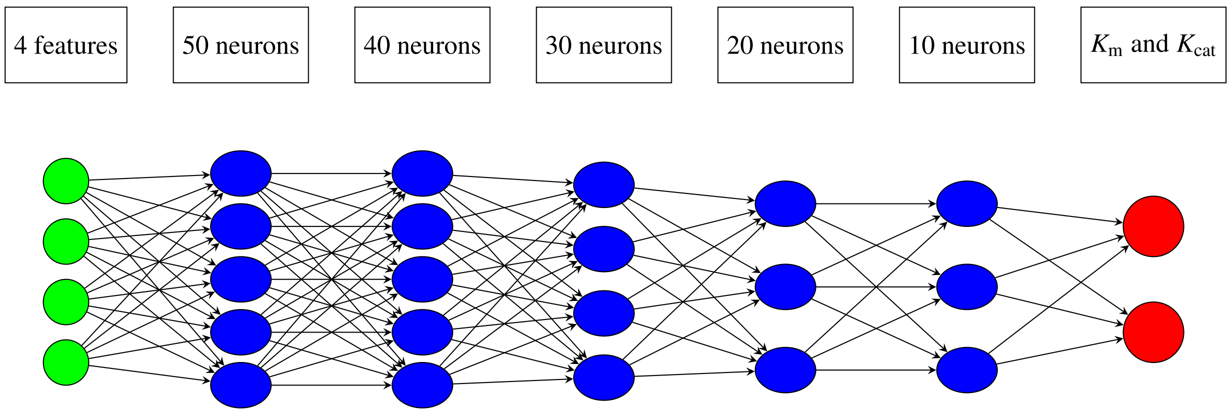

Figure S3: The architecture of the fully connected deep neural network (DNN) comprises input features (green), hidden layers (blue), and output labels (red). The input features include enzyme components (HRP and heme), temperature, substrate concentration, and pH values. The output labels correspond to the Michaelis-Menten (MM) parameters, K_m and K_cat.

7. The network was trained on preprocessed data, with weights saved for future use or fine-tuning.

8. Model performance was assessed using RMSE and regression analysis, with the Adam optimizer tuning parameters for accurate predictions.

References

[1] B. Choi, G. A. Rempala, J. K. Kim, Beyond the Michaelis-Menten equation: Accurate and efficient estimation of enzyme kinetic parameters,Scientific Reports 7 (1) (2017) 17018.

[2] S. Cha, Kinetic behavior at high enzyme concentrations: magnitude of errors of Michaelis-Menten and other approximations, Journal of Biological Chemistry 245 (18) (1970) 4814–4818.

[3] N. Noii, A. Khodadadian, J. Ulloa, F. Aldakheel, T. Wick, S. Francois, P. Wriggers, Bayesian inversion with open-source codes for various one-dimensional model problems in computational mechanics, Archives of Computational Methods in Engineering 29 (6) (2022) 4285–4318.

[4] Rogers, A., Gibon, Y. (2009). Enzyme Kinetics: Theory and Practice. In: Schwender, J. (eds) Plant Metabolic Networks. Springer, New York, NY. $10.1007/978-0-387-78745-9_4$

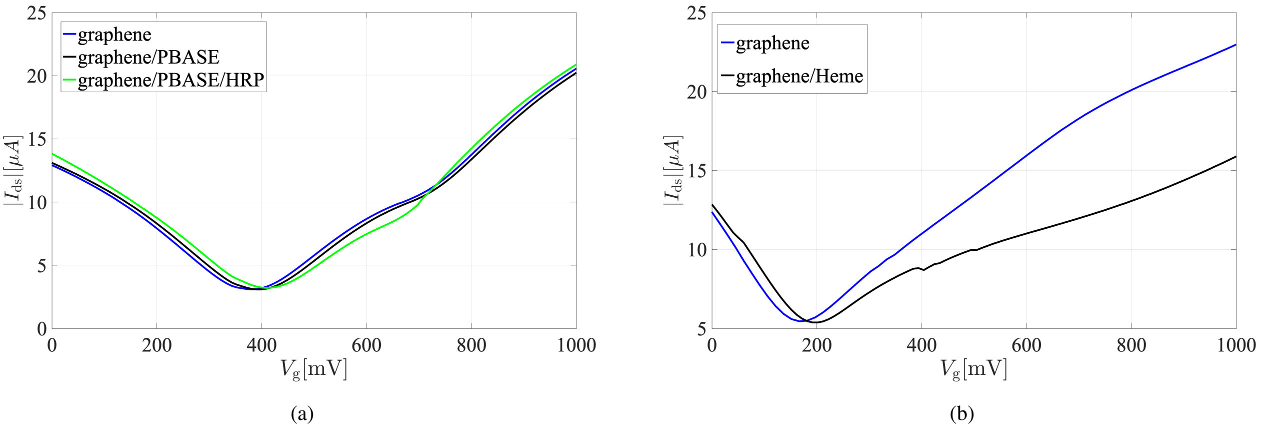

Figure S4: Transfer curve response corresponding to various stages of graphene surface modification: PBASE and HRP modifications in pH=7.4 (a) and Heme modification in pH=4 (b).

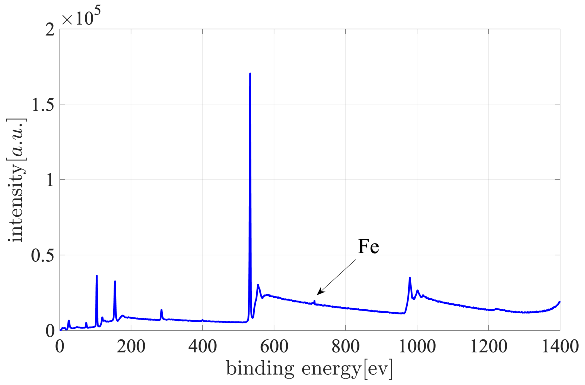

(a)

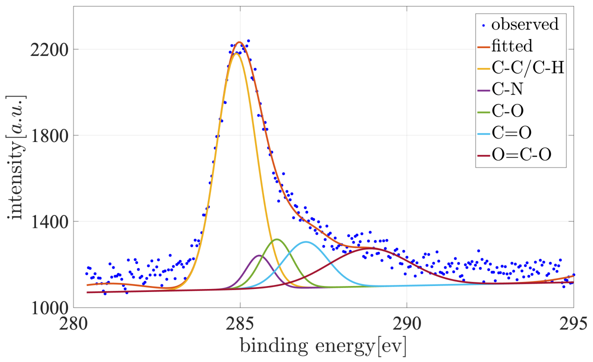

(b)

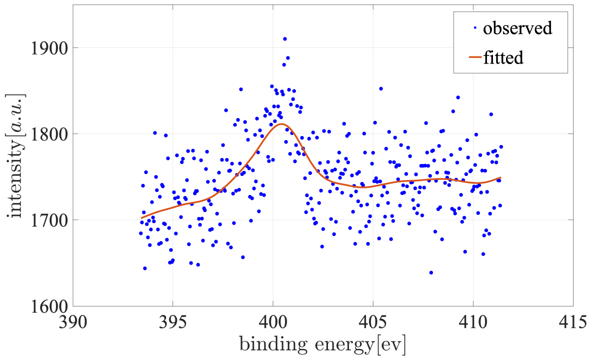

(c)

Figure S5: (a) XPS survey spectrum, (b) high-resolution C1s spectrum, and (c) high-resolution N1s spectrum for the Heme/Graphene. A cubic spline method is employed for data fitting.

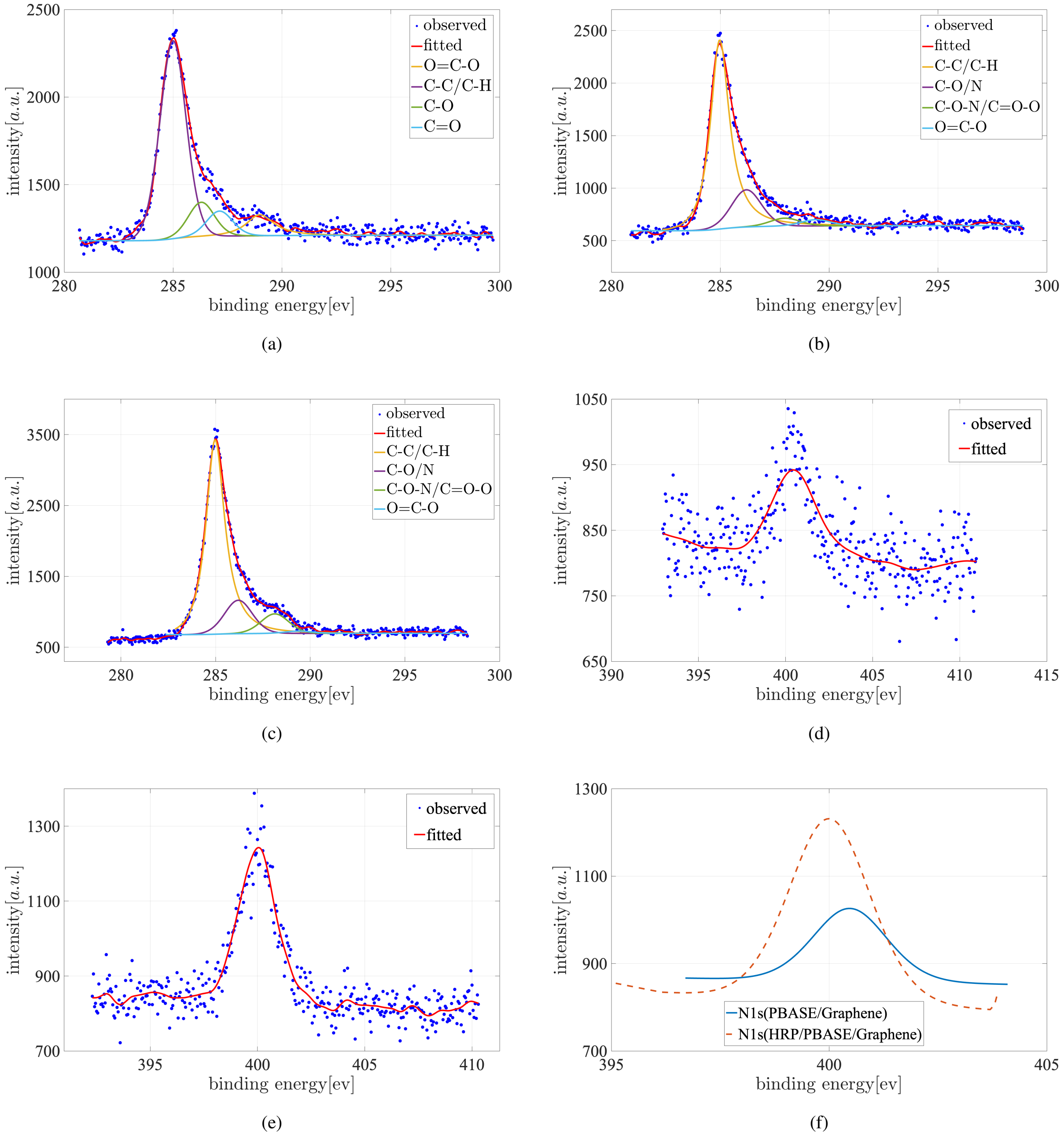

Figure S6: High-resolution XPS spectra of the C1s core level are presented for (a) Graphene, (b) PBASE/Graphene, and (c) HRP/PBASE/Graphene, alongside high-resolution XPS spectra of the N1s core level for (d) PBASE/Graphene and (e) HRP/PBASE/Graphene. The N1s peak (f) provides confirmation of the successful biofunctionalization of HRP on the PBASE/Graphene surface. Data fitting was performed using the cubic spline method.

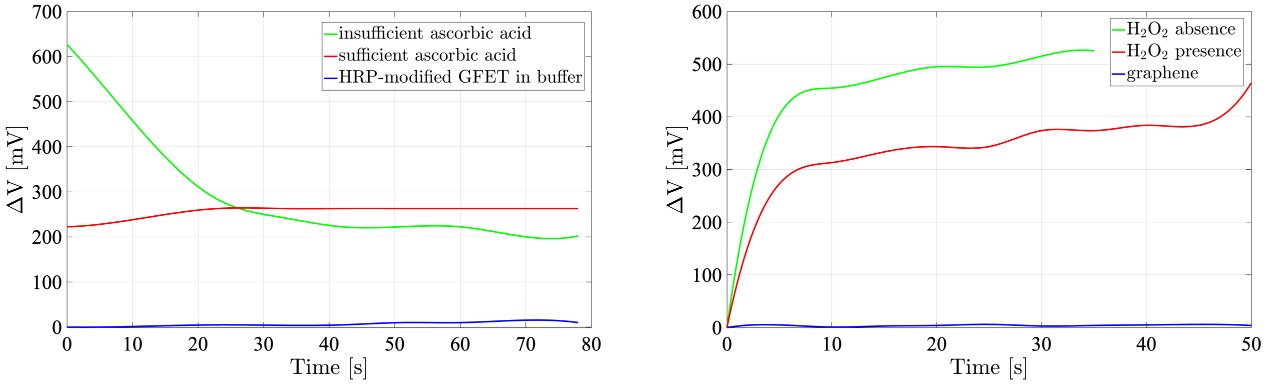

(a) (b)

Figure S7: Shift in Dirac voltage as a function of time for: (a) HRP-modified GFET in buffer (blue), in the presence of 50 μM H_2O_2 and 75 μM ascorbic acid (AA) (green), and in the presence of 50 μM H_2O_2 and 500 μM AA (red). (b) Heme-modified GFET before surface modification (blue), after Heme modification in the absence of H_2O_2 (green), and after Heme modification in the presence of 100 μM H_2O_2 (red).

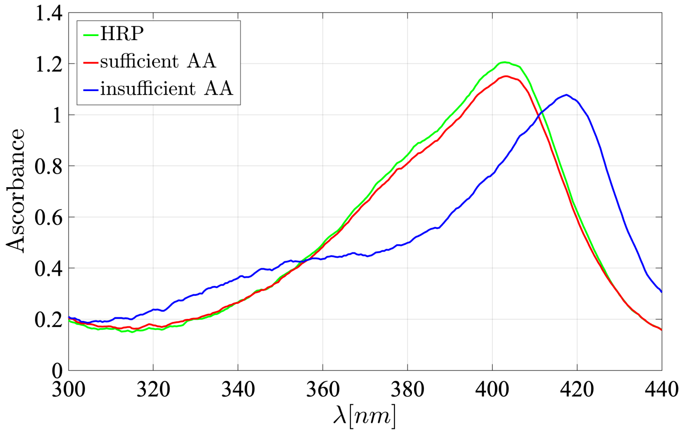

Figure S8: Soret band spectra corresponding to the HRP in buffer (green), HRP in the presence of 50 μM H_2O_2 and 500 μM AA (sufficient AA) and HRP in the presence of 50 μM H_2O_2 and 75 μM AA (insufficient AA).

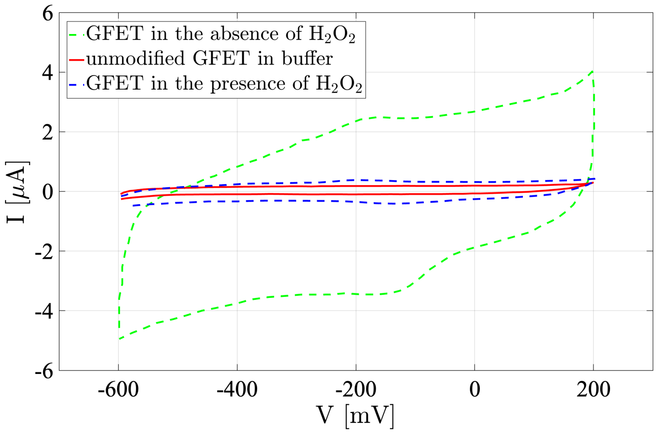

Figure S9: Three electrode CV for unmodified GFET in buffer (red curve), Heme modified GFET in the absence of H_2O_2 (green) and Heme modified GFET in the presence of 100 μM (blue). The scan rate is 50 mV/s.

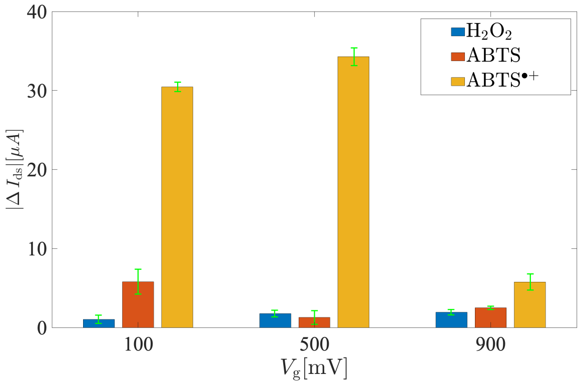

(a)

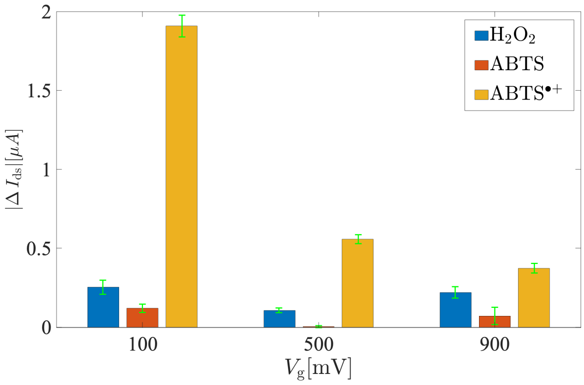

(b)

Figure S10: I_{ds} measurements in response to $100\,\mu$M of H_2O_2, ABTS, and $ABTS^{\bullet+}$ were conducted for HRP-modified GFET (a) and Heme-modified GFET (b). The devices were operated at varying V_g of 100 mV, 500 mV, and 900 mV, with a constant V_{ds} = 500 mV. The results are presented with error bars to indicate the deviations from the expected values.

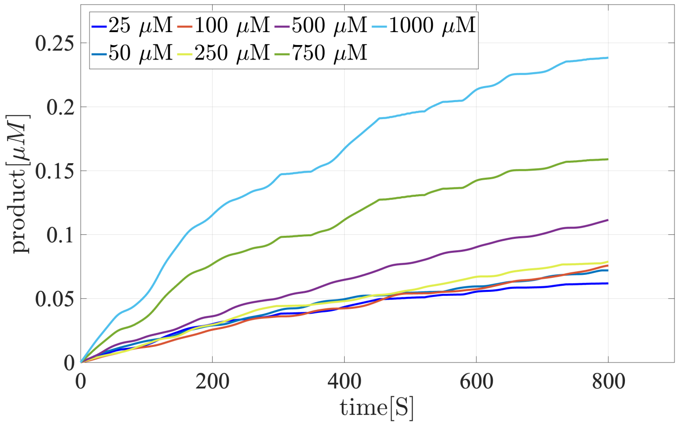

Figure S11: The HRP product rate over time for pH 7 for H_2O_2 different substrate concentrations.

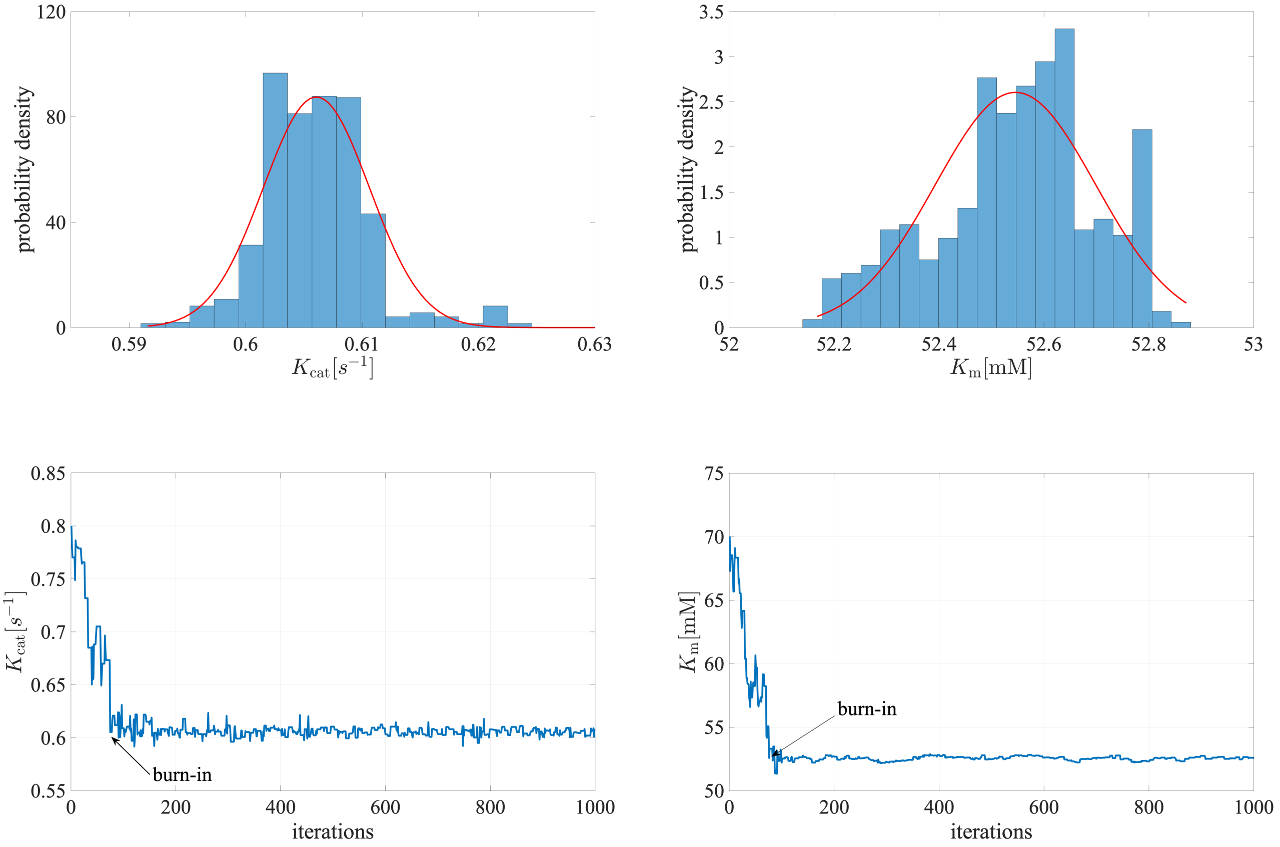

Figure S12: Top panels display the normalized histograms of the MCMC samples for the parameters K_{cat} (left) and K_m (right) after the burn-in period, illustrating their respective posterior distributions. The red line represents the fitted normal probability density function (PDF) for comparison. Bottom panels show the corresponding MCMC chains for K_{cat} (left) and K_m (right), providing insights into the sampling behavior and convergence over iterations.

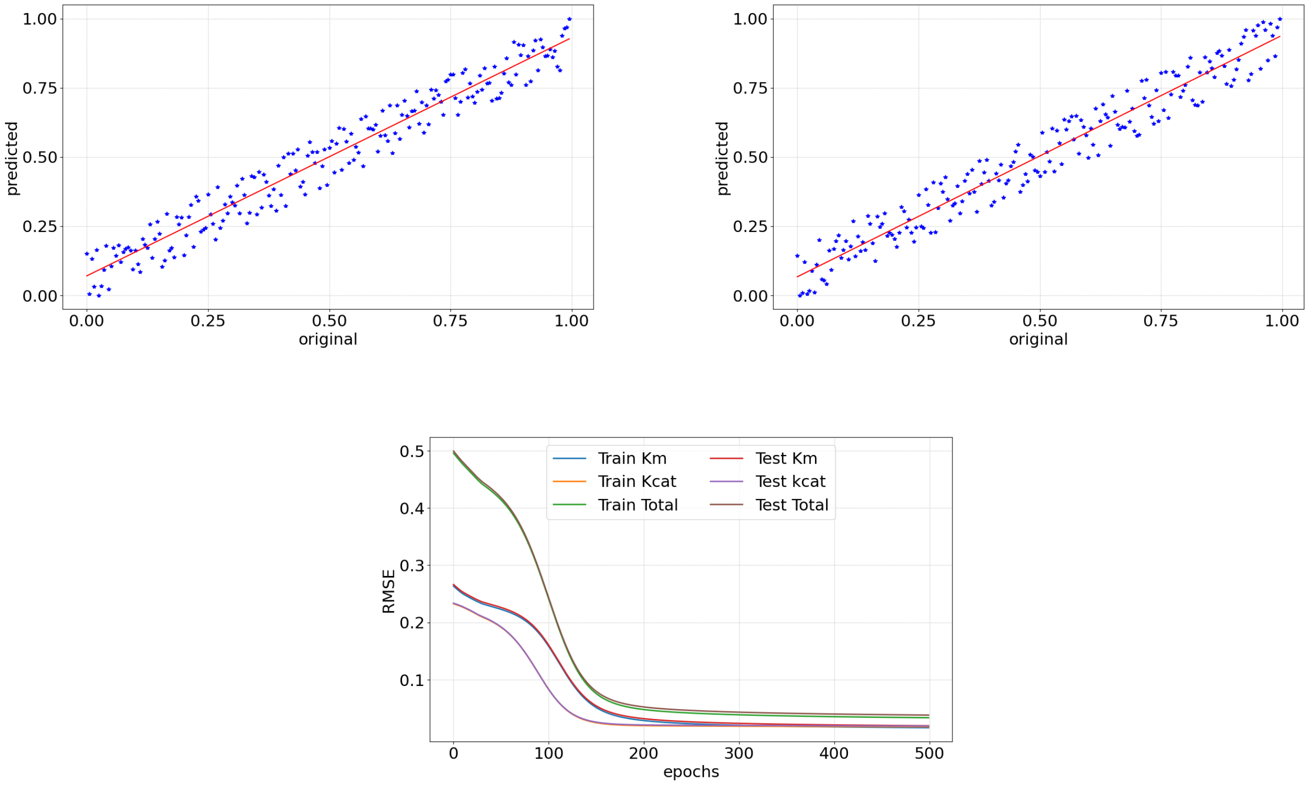

Figure S13: The top panels present the regression plots for K_{cat} (left) and K_{M} (right) in the context of HRP with H_2O_2 at pH 7 and a temperature of 25°C. The bottom panel illustrates the RMSE decay over epochs for the predicted values of K_{M} and K_{cat}, highlighting the model's performance improvement during training.

Compound	Substrate	pH	K_m [mM]	K_{cat} [s^{-1}]	Reference DOI
HRP	H_2O_2	5.75	0.022		10.1021/bi034609z
HRP	ABTS	5.75	0.88	890	10.1021/bi034609z
HRP	ABTS	4.5	0.33	10.1	10.1074/jbc.M406374200
HRP	ABTS	7	0.64	45.5	10.1021/bi001150p
HRP	ABTS	5	0.73 (0.2)	1.12 (0.15)	10.1039/c7dt01144j
HRP	ABTS	7	10.4 (0.8)	0.50 (0.05)	10.1039/c7dt01144j
HRP	ABTS	5	0.966	1.44	10.1002/bit.25483
HRP	ABTS	5	0.464	186.2	10.1016/j.enzmictec.2015.04.012
HRP	ABTS	5	0.19		10.1021/jf904431t
HRP	ABTS	5	0.22		10.1021/ja00132a003
HRP	H_2O_2	5	3.7	3.48×10^3	10.1038/nnano.2007.260
HRP	TMB	5	0.434	4.00×10^3	10.1038/nnano.2007.260
HRP	Pyrogallol	7.4	0.81	29.17	10.1002/chem.200305692
HEME	Pyrogallol	7.4		0.04	10.1002/anie.201108400
HEME/ Graphene	Pyrogallol	7.4	1.22	4.1	10.1002/anie.201108400
HEME/ Graphene	*H_2O_2*	*4*	*78.1*	*0.48*	
HEME/ Graphene	*H_2O_2*	*7*	*82.2*	*0.78*	
HEME/ Graphene	*ABTS*	*4*	*47.5*	*0.26*	
HEME/ Graphene	*ABTS*	*7*	*63.6*	*0.39*	
HRP	*H_2O_2*	*7*	*52.5*	*0.605*	

Table S1: Enzymatic parameters for HRP and heme under various conditions are presented, with references indicated. Our observations are highlighted in italic.
